# Challenges and Potential of Antibody–Drug Conjugates as Prospective Tuberculosis Therapeutics

**DOI:** 10.3390/microorganisms13102234

**Published:** 2025-09-24

**Authors:** Kenneth W. Foreman, Hui-Chen Foreman

**Affiliations:** 1 Department of Chemistry and Biochemistry, George Mason University, Fairfax, VA 22030, USA; kforema@gmu.edu; 2 Mercy LLC, Roswell, GA 30076, USA

**Keywords:** *Mycobacterium tuberculosis*, LAM, HspX, Ag85 complex, HBHA, LprG/P27, DnaK, KatG, Mpt64, SodA, GroES, PhoS1/PstS1, antibody-drug conjugates

## Abstract

Tuberculosis (TB), primarily caused by *Mycobacterium tuberculosis* (Mtb), remains a leading cause of infectious disease mortality worldwide. Global TB control efforts face several hurdles, including the lack of a broadly effective vaccine, limited sensitivity of current diagnostics, particularly for paucibacillary and extrapulmonary TB, and significant adverse effects associated with prolonged small-molecule drug regimens. The growing prevalence of multidrug-resistant (MDR) and extensively drug-resistant (XDR) strains further underscores the urgent need for innovative therapeutic strategies. We outline characteristics of next-generation TB therapeutics. We show that antibody (Ab)-drug conjugates (ADCs) satisfy many of those desirable characteristics. Since a major hurdle to this approach lies in Mtb-specific Abs, we highlight an open-access resource comprising a broad panel of Mtb-specific mouse monoclonal antibodies targeting key factors involved in Mtb survival, immune evasion, and pathogenesis. These critical Mtb virulence factors include heat shock proteins (GroES, DnaK, and HspX), surface-associated or secreted proteins (LAM, Ag85, HBHA, Mpt64/CFP-21, and PhoS1/PstS1), cell wall/envelope-associated proteins (LprG/p27), and detoxifying enzymes (KatG and SodA). The resource provides full-length sequences of the immunoglobulin variable regions, enabling antibody engineering and facilitating translational TB research across vaccine design, diagnostic development, and immunotherapeutic applications, in addition to ADCs. This ADC targeted delivery strategy holds promise for overcoming TB heterogeneity and eliminating both active and dormant Mtb populations within a single therapeutic formulation and offers a novel avenue for precision TB treatment.

## 1. Introduction

Tuberculosis (TB), a chronic infectious disease, ranks among the leading causes of infectious disease-related mortality and morbidity in human history [1,2,3]. TB, a respiratory disease transmitted via aerosol, disproportionately affects resource-limited and densely populated regions like South-East Asia and Africa [4,5]. Although the World Health Organization (WHO) has designated TB as a critical global health priority, the disease still affects more than 2 billion individuals worldwide [6,7].

*Mycobacterium tuberculosis* (Mtb), among the members of the *Mycobacterium tuberculosis* complex (MTBC), is the primary etiological agent of human TB [3,8]. Mtb is transmitted via aerosolized droplets and establishes infection in the lungs following phagocytosis by alveolar macrophages [9,10,11]. Mtb inhibits phagosome–lysosome fusion and maturation, thereby evading macrophage-mediated inactivation/degradation [10,11,12]. Mtb pathogenesis centers around its ability to survive and persist within these immature phagosomes [10,11]. Consequently, macrophages, rather than clearing the pathogen, become a reservoir for Mtb persistence [10].

The pathogenesis of TB is highly complex, shaped by dynamic interactions between host immune responses and pathogen-driven mechanisms [13]. Hosts, once infected, inherently struggle to clear Mtb. Mtb triggers an acute immune response during primary infection, reinfection, or reactivation that frequently transitions into a latent, chronic, or persistent state, from which reactivation may subsequently occur [3,13,14,15]. Although pulmonary TB is the most prevalent form, disseminated or extrapulmonary TB can involve lymph nodes, bones, or multiple organs [16]. The diverse and heterogeneous clinical manifestations [7,17] of TB complicate timely, sensitive, accurate diagnosis and thereby effective intervention [18].

Current TB intervention relies predominantly on combination therapies involving small-molecule antibiotics (SMDs) [19,20,21]. Typically, TB treatment schedules last for months, increasing the risk of considerable adverse effects [22]. Standard therapy for drug-sensitive TB includes four drugs: rifampin, isoniazid, pyrazinamide, and ethambutol for two months (the intensive phase), followed by rifampin and isoniazid for an additional four months (the continuation phase) [20,21,23,24]. Under specific conditions, a shorter four-month regimen employs rifapentine and moxifloxacin [19,24]. Treatment of MDR TB involves more complex regimens, such as the BPaL combination—bedaquiline (B), pretomanid (Pa), and linezolid (L)—administered for at least six months [19,25,26,27]. However, linezolid treatment features severe adverse effects, including hematologic toxicity, peripheral neuropathy, myelosuppression, and optic neuritis [26].

Mtb antibiotic recalcitrance arises from its slow growth rate, high adaptive resistance, phenotypic plasticity, and capacity to form biofilms, necessitating prolonged therapeutic regimens [14,28]. Within granulomas, Mtb can transition into a metabolically dormant, non-replicating state that enables long-term persistence [3,29]. This dormant phenotype renders conventional antibiotics largely ineffective, as most antimicrobials target actively dividing bacteria [29,30]. The limited repertoire of effective SMDs against the dormant phenotype represents a growing threat, particularly in the face of the global emergence of MDR-TB and XDR-TB strains [7].

Latent tuberculosis infection (LTBI) presents distinct challenges for disease control [3,31,32]. Reactivation of LTBI can account for more than 80% of active TB (ATB) cases in low-incidence countries [7,17,32]. Currently, any one of three treatment regimens is recommended for LTBI management [33]: (1) 3 months of once-weekly isoniazid plus rifapentine (3HP), (2) 4 months of daily rifampin (4R), and (3) 3 months of daily isoniazid plus rifampin (3HR). Although shorter and more tolerable than traditional six- to nine-month therapies, these regimens remain impractical for large-scale implementation, particularly in resource-limited settings, and fail as a stand-alone strategy for effective epidemic control.

TB prevention remains limited, as natural infection does not generate durable, protective immunity [11]. Moreover, the century-old Bacillus Calmette–Guérin (BCG) vaccine, the only current long-term preventive vaccine for TB, offers limited and variable protection, particularly in adults [11,13,34]. Most TB vaccine candidates remain in investigational stages of development [10,11,35,36]. The limited therapeutic arsenal currently available highlights the urgent need for intensified research and development for TB control.

Effective TB control requires an integrated approach involving improved diagnostic accuracy, expanded therapeutic options, and effective preventive strategies. Antibodies (Abs) have long served as essential tools in combating infectious diseases, contributing significantly to both therapeutic interventions and diagnostic innovations [37,38,39,40]. They exhibit multimodal functionality: the variable region (Fab or immunoglobulin variable [IgV] domain) mediates antigen (Ag) recognition, while the constant region (Fc) facilitates immune effector functions such as complement activation and engagement with Fc receptors on immune cells [41].

In this review, we highlight the critical features of next-generation TB therapeutics and a lesser-discussed yet promising therapeutic strategy: *Mycobacterium tuberculosis*-specific antibody–drug conjugates (MtbADCs). While this possibility was considered quite speculatively more than a decade ago, many new developments, particularly around Ab resources, warrant a deeper dive [42]. We discuss how MtbADCs fulfill many of the requirements of a next-generation TB therapeutic. Additionally, we discuss some of the issues that must be overcome for the successful implementation of this strategy, including proper Ab identification. An open-access antibody resource [43] is highlighted for its relatively recent verification of a broad panel of Mtb-specific monoclonal antibodies (mAbs) with functional IgV sequences. These Abs target key virulence factors, including surface antigens, important for Mtb survival and pathogenesis. We conclude with possible basic and translational research avenues enabled by the antibody resource.

## 2. Critical Features of Next-Generation TB Therapeutics

To elicit effective killing, local drug concentration at the site of infection and the bactericidal potency over time is crucial. Currently, targeted intervention strategies against Mtb remain limited. Next-generation TB therapeutics should demonstrate at least one of the following features:

- TB drugs should maintain robust bactericidal activity across diverse metabolic states, including Mtb variants with phenotypic heterogeneity and adaptive resistance.
- TB drugs must overcome the pharmacokinetic (PK) barriers of passive diffusion in reaching tissue or cellular barriers, e.g., granuloma and intracellular compartmentation.
- TB drugs must act as a potent, pan-TB regimen with improved pharmacodynamic (PD) and PK profiles relative to current therapeutics.
- Increased efficacy translates into regimens with shorter treatment duration relative to current therapeutics.
- TB drug regimens would preferably employ a single-agent therapy. Such simplification should improve patient adherence and enhance the operational feasibility of large-scale implementation in global public health programs.

## 3. Rationale for MtbADCs

**Figure 1:**
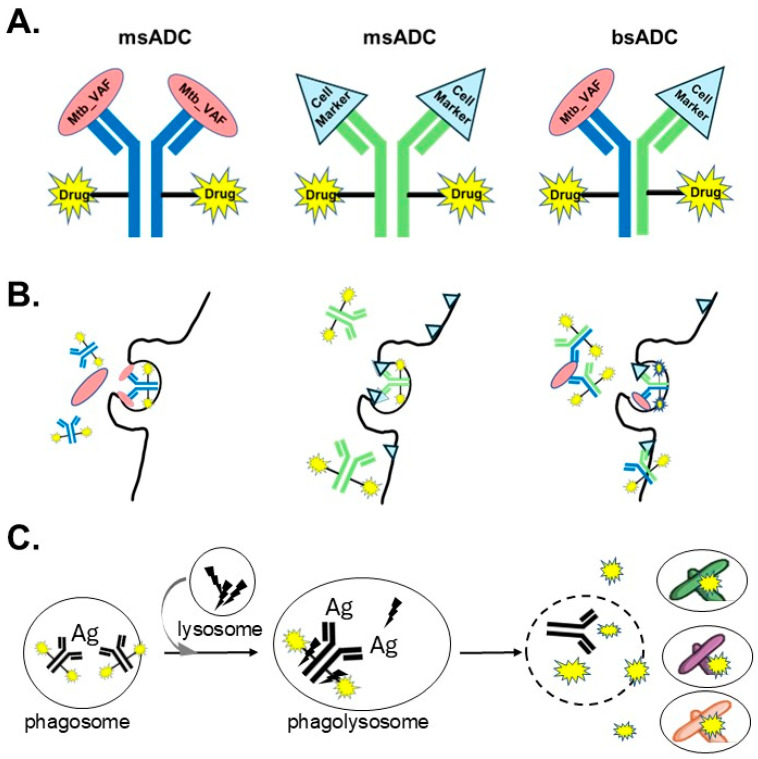
Schematic representation of the essential components of the *Mycobacterium tuberculosis*-targeting antibody drug conjugate (MtbADC) (**A**), its mechanism of internalization into macrophages (**B**), and its pharmacological mechanism of action against intracellular *Mycobacterium tuberculosis* bacilli (**C**). (**A**) MtbADC comprises an Ab, a cleavable linker (Black bar) and payloads (“Drug”, Yellow star). The Ab may be either monospecific (msADC) or bispecific (bsADC) and might recognize surface antigens of virulence-associated factors (Mtb_VAF, Pink oval, (**Left**)), cellular markers (Blue triangle, (**Middle**)) or both (**Right**). Payloads are frequently antimicrobials. (**B**) MtbADC mechanism of internalization. (**Left**) Opsonization. The Ab in the MtbADC binds the Mtb_VAF surface antigens. (**Middle**) The Ab in the MtbADC binds to antigens on the macrophage cell surface, forming a multivalent immunocomplex. (**Right**) Abs demonstrate greater versatility, with the capacity to both opsonize and recognize macrophage surface antigens. In all cases, FcγR receptor binding recruits actin machinery, inducing receptor-mediated phagocytosis. (**C**) MtbADC mode of action. The phagolysosomes form as the phagosomes containing the MtbADC-Ag complex fuse with lysosomes enriched in degradative enzymes such as cathepsins. In the presence of lysosomal enzymes, the ADC-linker degrades, resulting in controlled release of the payloads. Payloads are generally membrane-permeable, allowing access to other compartments in the cell once released. Mtb-killing modes include (1) directly acting on the novel, internalized population (Active replicating bacilli from ATB, Purple), and (2) Bystander effect, indirectly targeting the pre-existing or long-hidden latent, intracellular populations (Mtb phenotypic and metabolic variants, Green bacilli during both ATB and LTBI; Mtb persisters such as dormant bacilli, Orange bacilli during LTBI).

ADCs comprise the following components (Figure 1A) [44,45,46]:

- Payload: the bioactive agent or agents responsible for the therapeutic effect.
- Ab: a targeting moiety for precise delivery and a biodegradable carrier for the therapeutic payload.
- Linker: a chemical entity that covalently attaches the payload to the Ab, ensuring stability during circulation and controlled release primarily at the target site.

Rationally engineered MtbADCs can satisfy the requirements for next-generation TB therapeutics. Figure 1B,C illustrates the MtbADC conceptual framework.

### 3.1. MtbADCs’ Potential for Robust Bactericidal Activity

ADC targeted approaches have successfully treated intracellular infections, delivering antimicrobial agents directly into otherwise inaccessible compartments [47]. For instance, recent studies employing ADC-based antibiotic delivery against phagosome-resident methicillin-resistant *Staphylococcus aureus* (MRSA) showed substantial efficacy [48,49,50]. This “baiting” strategy, whereby cytotoxic or antimicrobial agents are actively directed into intracellular niches, appears feasible.

Ab–Ag binding is largely independent of pathogen metabolic state or intracellular localization, provided the ADC can access the target antigen. Therefore, Abs that target surface-exposed Mtb antigens conserved and consistently expressed across diverse infection states and phenotypic variants or that target inflammatory markers on macrophages could form the basis of broad-spectrum ADCs (Figure 1B). Once delivered to the Mtb, the ADC payload will have the opportunity to kill effectively due to the concentration enhancement at the point of infection (Figure 1C).

### 3.2. MtbADCs’ Potential to Overcome Passive Diffusion Limitations

Systemic passive diffusion affects the tissue distribution of traditional SMD therapies [51]. Necrotic and structurally complex tissues, such as TB granulomas, feature markedly inefficient passive diffusion to the therapeutic target [52,53,54]. TB granulomas can have highly organized structures with a complex microenvironment, including a central necrotic core (caseum) surrounded by concentric layers of infected and uninfected macrophages, T cells, fibroblasts, and fibrotic collagen [3,55,56]. While granulomas play a central role in the immunological containment of Mycobacterium, their dense architecture and heterogeneous microenvironment present significant barriers to SMD penetration and limit drug bioavailability at the site of infection [56]. While efficient Ab delivery depends heavily on circulatory access when delivered via intravenous or subcutaneous routes, they bind essentially irreversibly once they find their target and hence can accumulate over time. Due to their longer circulating half-lives, the need for significantly higher dosing remains a more remote possibility. We discuss this aspect in more detail in Section 6.

### 3.3. MtbADCs Hold Strong Potential as Pan-TB Agents

Pan-TB recognition requires antibodies that target surface antigens consistently expressed across diverse bacterial metabolic states and, ideally, conserved throughout the MTBC. Open-access antibody resources facilitate systematic screening to identify Mtb-specific antibodies against abundant, conserved surface antigens, establishing a foundation for broad-spectrum therapeutic strategies.

Furthermore, MtbADCs can be engineered for intracellular drug delivery, specifically targeting Mtb reservoirs in macrophages. When combined with optimized Fc-mediated effector functions, such ADCs could improve drug bioavailability within hard-to-reach intracellular compartments, supporting the development of a potent, pan-TB therapeutic regimen effective against both ATB and LTBI (Figure 1). We discuss these aspects more in Section 6.

### 3.4. MtbADCs Support the Development of Potent TB Therapeutics with Shorter Treatment Duration: Linker Function

In ADC design, the linker plays a critical role in ensuring payload stability in circulation while enabling controlled drug release at the target site. By providing safeguards against systemic release, linkers permit the use of highly potent but non-specific cytotoxic SMDs. Either a cleavable or an uncleavable linker can fulfill the goal of stable payload retention during systemic circulation [44,57]. Controlled drug release, traditionally either via pH change or enzymes acting only at the target site, minimizes unwanted cytotoxicity and maximizes the precision of drug action via localized, concentrated drug delivery. Such targeted delivery should result in greater kill rates in the main Mtb reservoir, thereby reducing necessary treatment schedules. We discuss these aspects more in Section 5.

## 4. Payloads in MtbADCs

Current TB treatment relies on multi-drug combination regimens to enhance therapeutic efficacy and minimize the emergence of drug resistance. Frontline agents such as isoniazid, rifampicin, and/or moxifloxacin can serve as ADC payloads. These inhibitors are incompletely buried when bound to the target active site—solvent still contacts some inhibitor atoms. These solvent-exposed atoms presumably confer little binding affinity to the inhibitor-target complex. Modification of these atoms can create handles from which linkers to the Ab may be attached without significantly altering the binding affinity of the complex. Note, however, that modifying an SMD can affect its PK/PD profile, and the modified product that the ADC releases must be profiled to ensure it behaves at least as well as the unmodified original. In addition to current frontline agents, more potent or less conventional TB agents can also be considered by leveraging the safety afforded by ADC linker technology. For example, early-stage candidates with otherwise limiting toxicities, such as thioridazine (TZ), could be repurposed. TZ exhibits broad-spectrum antimycobacterial activity against drug-sensitive and drug-resistant intracellular Mtb [58,59], but cardiotoxicity and neurotoxicity constrain its clinical use [60,61]. Incorporating TZ into an ADC formulation involving a linker designed for compartment-specific drug release could mitigate these off-target effects and increase therapeutic potential.

Enhancing the therapeutic potential of the payload, particularly in intracellular environments, remains an active area of research. For example, KRM-1657, a decades-old compound, demonstrated superior in vitro activity against Mtb compared to rifampicin [62]. Yet because of unacceptable side effects, its development was discontinued. Recently, KRM-1657 was incorporated into ADCs, showing prolonged activity against *Staphylococcus aureus* within phagosomes [50] with significantly reduced side-effects. Once again, the ADC approach to target intracellular pathogens appears viable while simultaneously mitigating otherwise harmful side effects.

## 5. Linkers for ADCs

Enzyme-cleavable linkers, such as the valine-citrulline (Val-Cit) dipeptide linker, susceptible to cathepsin B (CatB) activity, could offer particular benefits in the TB context. Cathepsin enzymes localize within phagolysosomes and cleave in a pH-dependent manner, with activity increasing as the macrophage phagolysosome acidifies. Payloads would theoretically release predominantly within the Mtb reservoir, minimizing off-target effects while preserving therapeutic potency and precision (Figure 1B). Yet, Mtb interrupts pH lowering in phagolysosomes, thus significantly lowering the rates of cleavage for most cathepsins. Some CatB activity still remains at pH 6.5, a typical acidity for the immature phagolysosome. As long as the payload cannot rapidly escape the local environment or can target both actively replicating and dormant bacilli, the risk of metabolic variants and persisters developing strong resistance should be low while allowing for significant kill rates over time.

Other, less traditional linkers may offer greater opportunities for specifically targeting Mtb-infected macrophages. Potential candidates include Cathepsin S (CatS)- or β-glucuronidase (BG)-cleavable linkers. Much like CatB, both CatS and BG are almost exclusively found in phagolysozomes, implying that the linkers will release their payloads almost exclusively in phagolysosomes. CatS still retains strong catalytic activity at or below pH 7 [63], making linkers that include its substrate a reasonable choice. The peptides Val-Cit and GGFG are known CatS substrates and effective linkers, particularly with self-immolating p-aminobenzyloxycarbonyl spacers (PABCs) proximally attached. Nevertheless, optimization towards CatS substrates at pH 7 would permit more efficient payload release in Mtb reservoirs. Similarly, BG-cleavable linkers have been frequently employed, with cleavage activating PABC self-immolation and release of the payload [46]. Since Mtb does not down-regulate BG activity, linkers incorporating its substrate (a β-glucuronide), together with a PABC spacer, appear as strong candidates in MtbADCs. Alternate linkers that do not rely on pH or enzymatic cleavage would likely need to rely on other enzymes, absent in circulation, but available in the immature phagolysosome and active at pH 6.5.

The flexible modularity of the ADC platform enables integration of diverse payload classes to achieve additive or synergistic effects. Advances in linker chemistry, including branched designs [57], enable ADCs to carry multiple bioactive agents as the payload, achieving additive or synergistic antimicrobial effects and accommodating novel therapeutic modalities beyond conventional SMDs. In addition to carrying bactericides or cytotoxic compounds, host tissue repair or immune modulators could be dosed in combination, for instance, with MtbADCs for host-directed therapy (HDTs) [64,65]. Alternatively, branched linkers carrying both antimicrobials and factors beneficial to the host could be attached to a single Ab as the payload [57]. These HDTs may serve to normalize the microenvironment of granulomas, enabling more effective ADC PD.

## 6. Mtb Antibody Resource for MtbADC Abs

### 6.1. Antibody Selection for MtbADC Development

In this review, we highlight a promising yet underexplored avenue in TB research, MtbADCs. While several potent SMDs exist and certain linkers are very well established, Mtb-specific Abs remain underexplored for viability as a component in an ADC, thus limiting an immediate MtbADC application. Ab selection critically determines the eventual success of the MtbADC. It determines affinity for either the Mtb or macrophage antigens, as well as selectivity for the macrophage phagolysosome over other tissues and other compartments. It strongly influences circulatory half-lives and, hence, favorable PK. The Biodefense and Emerging Infections Research Resources Repository (BEI, https://www.beiresources.org/, accessed on 28 August 2025), an open-access Mtb Ab resource, has significantly advanced opportunities employing this approach in the past few years. This resource stems from a National Institute of Allergy and Infectious Diseases (NIAID) initiative aimed at supporting TB research and clinical development. BEI produces, curates, and distributes mouse monoclonal Abs derived from deposited hybridoma clones raised against key Mtb virulence factors involved in bacillary pathogenesis and immune evasion [43,66]. The BEI Mtb Abs can be screened for surface-exposed Mtb native Ags conserved and consistently expressed across diverse infection states and phenotypic variants. Viable hits would serve as a backbone for ADC construction.

The antigen repertoire spans both surface-associated and secreted proteins, including lipoarabinomannan (LAM) [67,68,69], the Ag85 complex [70], heparin-binding hemagglutinin (HBHA) [71], Mpt64/CFP-21 [72], and PhoS1/PstS1 [73]. It also includes cell wall or envelope components such as LprG/P27 [74], molecular chaperones including GroES [75], DnaK [76], and HspX [77], which contribute to bacterial fitness and immune modulation. Additional antigens include detoxification enzymes such as catalase-peroxidase (KatG) [78,79] and superoxide dismutase A (SodA) [80], critical for Mtb resistance to oxidative stress within host phagosomes. Many of these antigens have been prioritized in recent years for TB intervention strategies, including vaccine development (e.g., Ag85, HspX, HBHA, and DnaK) [36,81,82,83,84], diagnostic tools (e.g., Mpt64, LAM, and KatG mutations) [85,86,87], and therapeutic approaches (e.g., LAM) [67].

Within this resource, surface antigens such as LAM, PhoS1/PstS1, HBHA, and LprG/p27 emerge as particularly promising targets. For instance, PhoS1/PstS1, requisite for Mtb virulence, functions as an ABC transporter involved in phosphate homeostasis [73]. Antibodies against PhoS1/PstS1 attenuate Mtb pathogenicity [88]. An ADC directed at this antigen could deliver the dual benefits of virulence suppression (via Ab) and pathogen killing (via payload drug). Importantly, PhoS1/PstS1 is a membrane-anchored, non-secreted protein, a favorable feature for precision targeting, and is also highly conserved across MTBC, with ~95% pairwise identity based on NCBI BLASTp analysis (https://www.ncbi.nlm.nih.gov/, accessed on 28 August 2025). An ADC targeting such an antigen could therefore represent a broad therapeutic strategy against both Mtb and related mycobacterium pathogens.

### 6.2. Ab Effector Function for MtbADC Development

Careful design can enable MtbADC binding to either infected macrophages or bacilli-associated Ags, triggering the uptake into efficient endocytic/phagocytic or receptor-mediated pathways. Mtb internalization in professional phagocytes (such as macrophages) is primarily mediated via phagocytosis, which antibody- and complement-mediated opsonization strongly enhance [89]. In addition to the classical Fcγ receptor-mediated phagocytosis (Figure 1B,C), bacilli can also enter cells through other routes, such as receptor-mediated endocytosis (e.g., mannose receptor [90]; scavenger receptors [91] or macropinocytosis [92]). Figure 1 shows the major pathogen internalization route and suggests possible antibody engineering can be accompanied to enforce the endocytic pathway. For instance, to increase internalization and ensure specificity, the antibody could be engineered as a bispecific Ab (Figure 1A, Right) eliciting greater versatility with one arm binding to an Mtb surface Ag and another arm recognizing infection-upregulated macrophage markers, e.g., FcγRI (CD64) [93] or mannose receptor (CD206) [90,93]. This dual valency may improve endocytosis/phagocytosis efficiency, increasing the frequency of payload cleavage in phagolysosomes.

MtbADCs will benefit from additional functionalities. Mtb can partially block fusion of the phagosome with the lysosome (“fusion event”) and then indefinitely forestall phagolysosome maturation via, e.g., SapM, ManLAM, PtpA, or Ndk [94,95,96,97]. Fc-engineering for high-affinity FcγRI/II binding becomes important to counteract these functions. FcγR-mediated phagosomes have higher rates of fusion with lysosomes compared to complement receptor–mediated phagosomes [98,99,100]. Strong FcγR crosslinking can create sustained signaling through the Syk/PI3K/PLCγ pathway, leading to actin remodeling that at least partially overcomes coronin-1/ManLAM-mediated arrest of fusion events [98]. Combination therapeutic approaches represent a viable alternative. In addition to the MtbADC, host-directed modulators such as the PknG inhibitor AX20017 [101] or immune modulators such as drugs that boost Ca^2+^ signaling [102] may restore phagosome maturation and help ADCs reach lysosomal compartments to release their payload.

The BEI resource holds particular value in Fc-engineering, providing the corresponding IgV or Fab sequences for many Mtb-targeting Abs, thereby facilitating Fc isotype switching through recombinant antibody engineering. These sequences could readily and critically assist in developing antibody-based therapeutics with improved specificity, sensitivity, half-life, and safety, or advance fundamental antibody studies of TB immunity. For instance, Grace P.S. et al. recently utilized sequence data from this resource to show that Fc-effector function differentially contributes to early Mtb restriction [103].

Table 1 describes the Mtb-Ab collection and Supplementary S1 and Tables S1 and S2 list their corresponding GenBank accession numbers and their sequence information, including their framework regions (FR1–FR4) and the complementarity-determining regions (CDR1–CDR3) of the IgV sequence. Domain annotation follows the numbering schemes of IMGT (https://www.imgt.org/HighV-QUEST/http://www.imgt.org/IMGT_vquest/vquest, accessed on 19 September 2025) (Table S1) or KABAT (https://www.ncbi.nlm.nih.gov/igblast/igblast.cgi, accessed on 28 August 2025) (Table S2).

This BEI resource provides high-fidelity sequences for the clones in Table 1. Hybridoma sequencing frequently yields multiple antibody sequences, including incomplete or nonfunctional variants [104], and many publicly available IgV (Fab) sequences lack verification for productive immunoglobulin expression or epitope-binding. In contrast, this resource employs a deep sequencing approach to identify antibody-encoding IgV regions from hybridoma clones. To ensure functional relevance, Ab–Ag interactions were validated through epitope-binding assays using an isotype-switching approach [43]. Only verified and functional IgV sequences were deposited into GenBank, ensuring a reliable foundation for downstream applications.

Despite the favorable developments in Ab selection, experimental determination of the penetration rate and whether such a rate could deliver sufficient doses of their payloads to Mtb remains a crucial unknown. Antibody engineering has advanced significantly in recent years [46], enabling smaller sized ADCs [105] that, e.g., employ a monovalent antibody to enhance tissue penetration. Ab half-life [106] and phagocytotic capacity [107] can be enhanced to increase drug accumulation at the desired target. Combination strategies with other therapeutics, such as HDTs, to improve the granuloma microenvironment may also be beneficial. The route of administration may provide a means to circumvent circulation barriers to some degree. For instance, employing an air inhaler for direct pulmonary exposure (in low-resource settings) or direct injection into granulomas (in clinical settings) should localize MtbADCs largely to target tissues. Ultimately, a successful ADC approach will depend on optimizing antibody access, internalization efficiency, and stability within the granuloma microenvironment to overcome the barrier of a poorly vascularized necrotic core.

Predicting ADC stability is more art than science, but empirically, half-lives in circulation range from hours to weeks [108]. Optimizing ADCs to exhibit longer serum half-lives will, in principle, assist in the targeted delivery of payloads to infection sites with improved PK and PD. Several of the ADC’s components impact their stability. For instance, the antibody types [45], the linker chemistry [109,110], the site of payload (SMD) attachment to the antibody [109,110], and the drug-to-antibody ratio (DAR) [110] all play a role. For standard IgG antibodies, the half-life of IgG1, IgG2, and IgG4 subtypes is approximately three weeks in blood, whereas for IgG3, it is around only one week [45]. Extending the half-life of the ADC may be possible via clever engineering [106]. Importantly, different clearance rates can occur between patient and control cohorts and should be accounted for during research and development stages [48,111,112].

## 7. Conclusions

The persistent global burden of Mtb-induced TB underscores the urgent need for transformative therapeutic strategies. Future inhibitors must hold bactericidal activity against both actively replicating and dormant bacilli. Further, these inhibitors must achieve sufficient tissue penetration. Effective inhibitors will reach Mtb persisters in recalcitrant environments such as heterogeneous lesion microenvironments or “reservoir” tissues that enable persistence or relapse (e.g., paucibacillary macrophages). Next-generation agents must also combine target specificity with high potency to mitigate systemic toxicity and reduce drug–drug interactions, particularly in populations with comorbidities such as HIV or diabetes. Although improved PK and PD properties represent significant milestones, shortening treatment duration remains the critical benchmark, as regimen length continues to drive poor adherence and subsequent emergence of resistance. Ultimately, a simplified, broadly active “pan-TB” therapeutic, effective across drug-susceptible, MDR, and XDR strains, represents a disruptive technology advance, streamlining clinical management and accelerating global TB elimination efforts.

MtbADCs align closely with the attributes required for transformative TB therapeutics. In addition to improving PK profiles relative to SMDs, antibodies provide a powerful dual function: Fab-mediated Ag recognition for precise targeting of Mtb virulence factors, and Fc receptor–mediated internalization to facilitate intracellular delivery. By leveraging high-affinity Ab–Ag interactions, MtbADCs offer potential to overcome the limitations of passive SMD distribution across all tissues and enable co-localized concentration of payloads into Mtb-infected tissues. This targeted delivery not only enhances PD properties but also raises the prospect of reducing treatment cycles compared with conventional regimens.

The selection of antibodies as the backbone of MtbADCs will ultimately determine their therapeutic scope, whether deployed as highly specific agents or as broad-spectrum pan-TB regimens. Open-access repositories such as the BEI Resources, NCBI (https://www.ncbi.nlm.nih.gov/, accessed on 28 August 2025), the European Nucleotide Archive (ENA, https://www.ebi.ac.uk/ena, accessed on 28 August 2025), and the AntiBodies Chemically Defined (ABCD) Database (https://web.expasy.org/abcd/, accessed on 28 August 2025) collectively provide a growing pool of TB-specific antibody sequences. However, a major limitation across many of these repositories is the lack of comprehensive experimental validation, particularly regarding expression, secretion, and epitope-binding. This gap restricts their immediate translational application, especially for ADC development, since these Abs require extensive expression and Ag-binding validation before considering incorporation into ADCs.

Nevertheless, the expanding deposits into Mtb Ab repositories constitute a critical enabling tool for TB research and innovation beyond ADCs. These resources provide valuable assets for biomarker discovery that could distinguish active from latent or dormant infection, thereby advancing early detection, disease staging, and precision clinical management. In parallel, these Abs support mechanistic investigations into TB immunity, including the humoral contributions that guide rational vaccine design. Collectively, Mtb Ab resources represent an indispensable foundation for both basic and translational research. By accelerating the development of improved diagnostics, therapeutics, and vaccines, they hold promise for overcoming current barriers and driving progress toward global TB control and eventual eradication.

A criterion for selecting the exact Ab for ADC deployment involves the abundance of the Ag recognized by the Ab, with preference given to more abundant Ags. The greater the abundance, the fewer payloads necessary per ADC. BEI’s curated library of Mtb antibodies directed against critical surface Ags offers the opportunity for pan-TB recognition by attenuating Mtb virulence while directing SMDs to Mtb with high specificity. Their abundance still requires quantification. This resource also provides ready access to Ab IgV sequences for down-stream engineering and optimization. Together, this resource and associated Abs enable the rational design and evaluation of MtbADCs as feasible candidates for TB therapeutics.

Recent advances in ADC linker technology further elevate the promise of this approach. Modern cleavable and highly stable linkers provide systemic safeguards against premature release while ensuring efficient payload liberation within infected cells. Moreover, the emergence of branched linker designs offers the possibility of incorporating multiple bioactive agents into a single payload construct, increasing the feasibility of a unified “one-regimen” TB therapy.

Many excellent SMDs are available as payloads. Selecting the Ab and linker, therefore, represents the greatest hurdle to realizing therapeutic MtbADCs. The relatively high pH (~6.5) found in the immature phagolysosomes of macrophages limits deployment of many of the more common ADC linkers, suggesting more innovation in this space may be required to achieve optimal delivery. Nevertheless, linkers that couple either CatS cleavable peptide bonds or β-glucuronides (BG substrates) with self-immolating PBMCs appear likely to succeed without significant modification.

One of the remaining great challenges lies in the delivery of ADCs. To treat pulmonary TB, concentrated, direct, local delivery would avoid systemic circulation; thus, increasing drug stability and enhancing patient safety. The drug formulation could be nebulized liquids or dry-powders and administered via a disposable inhaler. Dry powder offers thermostability and ease in handling, storage, and shipping. While these characteristics complement well the needs of community/home health-care providers in low resource areas, uniform preparation of intact and/or functional Abs in powder form remains an area of active research. This practical consideration must be overcome in order to make large-scale investments in Mtb-ADC research worthwhile. As a potential alternative, collaborations between governmental agencies, such as the WHO, with large pharmaceutical companies could enable delivery of viable ADCs using a nebulizer strategy.

For extrapulmonary TB, systemic delivery will be necessary. Long half-lives in circulation will become essential. The drug formulation should be suited for intravenous or subcutaneous routes of administration, hence excluding most low resource environments. Clinical, hospital, or other highly trained staff will likely be required for administration. Nevertheless, if successfully developed, this strategy holds promise to simplify TB treatment into a single-agent regimen, thereby improving patient adherence and advancing global TB control and eradication efforts.

Coupling Ab advances with ADCs could transform TB into a much more managed disease in the next two decades. A successful design should couple antibody targeting (via Ab selection and engineering), Fc-biology, linker chemistry, payload selection, and a feasible delivery route that maximizes macrophage access. Based on the success of therapeutic ADCs targeting tumors, ADC designs should exhibit high stability in blood, maintaining >90% intact conjugated payloads while circulating for days to weeks, until reaching the targeted compartments [109]. Employing linkers resistant to hydrolysis except in the phagolysosome largely achieves this profile. Well-engineered Abs serve to increase circulatory lifetimes while increasing target specificity. The ideal MtbADC, based on current standards, would consist of an antibody that recognizes a pan-Mtb Ag across clinical variants, with strong Ag-binding and efficient internalization into target cells, e.g., an anti-CD64/anti-PhoS1/PstS1 bispecific Ab. It should exhibit effective tissue penetration and a sustained serum half-life to enable drug accumulation at the site of infection, e.g., Fc-engineered for high-affinity FcγRI/II binding and dosed with HDTs that enable better granuloma penetration. The linker, such as a Val-Cit peptide cleaved by CatS, would ensure controlled drug release within the targeted compartment. The payload, for example, TZ, should provide broad-spectrum bactericidal activity against diverse Mtb phenotypes, including non-replicating bacilli. Finally, the formulation should allow for route-specific dosing, whether by pulmonary delivery or systemic circulation. MtbADCs are positioned as a transformative platform for potent single-agent TB therapeutics, as long as the issues in the engineering of the Ab can be overcome.

## Figures and Tables

**Table 1:** IgV sequenced BEI Mtb hybridomas and the corresponding antibody-associated information compiled from the BEI and NCBI websites. IgV sequences are deposited in NCBI GenBank. See the Supplemental Information for accession numbers.

BEI-Cell Culture Collection	Clone	Antigen	Target Gene	Corresponding BEI-Ab Item	Ab Class
NRC-2893	CS-35	LAM	n/a	NR-13811	IgG3κ
NRC-2895	CS-49	HspX	Rv2031c	NR-13814	IgG1
NRC-2897	CS-90	Ag85 complex	Rv3804c Rv1886c Rv0129c	NR-13816	IgMκ
NRC-2914	a-HBHA	HBHA	Rv0475	NR-13804	IgG2a
NRC-13806	a-Rv1411c	LprG/P27	Rv1411c	NR-55708	IgG1κ
NRC-49679	clone A	DnaK	Rv0350	NR-49679	IgG1κ
NRC-50100	clone B	DnaK	Rv0350	NR-50100	IgG1κ
NRC-50101	clone B	KatG	Rv1908c	NR-50101	IgMκ
NRC-50703	Anti-Mpt64, Clone A	Mpt64	Rv1980c	NR-59474	IgG1κ
NRC-13810	CS-18	SodA	Rv03846	NR-13810	IgG1κ
NRC-49680	clone A	KatG	Rv1908c	NR-49680	IgMκ
NRC-2894	IT-3 (SA-12)	GroES	Rv3418c	NR-49223	IgG2aκ
NRC-2410	IT-15 (TB72)	PhoS1/PstS1	Rv0934	NR-13605	IgG1κ

## Data Availability

No new data were created or analyzed in this study. Data sharing is not applicable to this article.

## Supplementary Materials

The following supporting information can be downloaded at: https://www.mdpi.com/article/10.3390/microorganisms13102234/s1. Table S1. Summary of the nucleic acid sequences of immunoglobulin light and heavy chains based on the IMGT definition. Table S2. Summary of the nucleic acid sequences of immunoglobulin light and heavy chains based on the KABAT definition. Supplementary S1. FASTA IgV (Fab) sequences for BEI-Mtb Hybridoma clones in Table 1.

## Abbreviations

The following abbreviations are used in this manuscript:

TB	Tuberculosis
Mtb	*Mycobacterium tuberculosis* or *M. tuberculosis*
LAM	Lipoarabinomannan
HspX	Heat shock protein X
Ag85	Antigen 85 complex
HBHA	Heparin-binding haemagglutinin
LprG	Lipoprotein regulatory G
SodA	Superoxide Dismutase A
KatG	Catalase–Peroxidase G
Mpt64	Mycobacterium protein 64
PhoS1/PstS1	Phosphate-binding protein S1
Ab	Antibody
Ig	Immunoglobulin
IgV	Immunoglobulin variable chain
CDR	Complementarity determined regions
FR	Framework regions
Fab	Fragment antigen-binding
Fc	Immunoglobulin constant fragment
