# Supplementary material for: Challenges and Potential of Antibody–Drug Conjugates as Prospective Tuberculosis Therapeutics
Source: Microorganisms. 2025 Sep 24;13(10):2234. doi: 10.3390/microorganisms13102234 (PMC12566324; doi:10.3390/microorganisms13102234)
Supplement: Supplementary file 1 [file microorganisms-13-02234-s001.zip › Foreman_MtbADC_IMGT_Table S1.pdf]

Table S1. Summary of the nucleic acid sequences of immunoglobulin light and heavy chains based on the IMGT definition

Hybridoma	Ig (Genbank #)	FR1	CDR1	FR2	CDR2	FR3	CDR3
**NRC-13806 (LprG/p27)**	**IgG1 (MW854314)**	CAGGTCCAACTGCAGCAGTCTGGGGCTGAACTGGTGAAGCCTGGGGCTTCAGTGAAGTTGTCCTGCAAGGCTTCT	GGCTACACCTTCACCAACTACTAT	ATGTACTGGGTGAGGCAGAGGCCTGGACAAGGCCTTGAGTGGATTGGAGAG	ATTTATCCTAGCAATGGTGGTACT	AACTTCAATGAGAAGTTCAAGAACAAGGCCACACTGACTGTAGACAAATCTTCCACCACAGCATACATGCAGCTCAGCAGCCTGACATCTGAAGACTCTGCGATCTATTAC	TGTACAACTCACCTTGCTTACTGG
	**Igκ (MW854315)**	GATGTTTTGATGACCCAAACTCCACTCTCCCTGCCTGTCAGTCTTGGAGATCAAGCCTCCATCTCTTGCAGATCTAGT	CAGAGCATTGTACATAGTAATGGAAACACCTAT	TTAGAATGGTACCTGCAGAAACCAGGCCAGTCTCCAAACCTCCTGATCTAC	AAAGTTTCC	AACCGATTTTCTGGGGTCCCAGACAGGTTCAGTGGCAGTGGATCAGGGACAGATTTCACACTCAAGATCAGCAGAGTGGAGGCTGAGGATCTGGGAGTTTATTAC	TGCTTTCAAGGTTCACATGCTCCGTACACGTTC
**NRC-2893 (LAM)**	**IgG3 (MW854316)**	GAGGTTCAGCTCCAGCAGTCTGGGACTGTGCTGGCAAGGCCTGGGACTTCCGTGAAGATGTCCTGCAAGGCTTCT	GGCTACAGCTTTACCAACTACTGG	ATGCACTGGGTAAAACAGAGGCCTGGACAGGGTCTAGAGTGGATTGGTTCT	ATTTATCCTGGAAATAGTGATACT	AACTACAAGCAGAAATTCAAGGGCAAGGCCAAACTGACTGCAGTCACATCCGCCAGCACTGCCTACATGGAGGTCAACAGCCTGACAAATGAGGACTCTGCGGTCTATTACTGT	ACAAGATTTGGTAACTACGTTCCGTTTGCTTAC
	**Igκ (MW854317)**	GATATCCAGATGACACAGACTACATCCTCCCTGTCTGCCTCTCTGGGAGACAGAGTCACCATCGGTTGCAGGGCAAGT	CAGGACATTGGCAGTTAT	TTAAACTGGTATCAGCAGAAACCAGATGGAGCTGTTAGACTCCTGATCTAC	TACACATCA	AGATTACACTCAGGAGTCCCATCAAGGTTCAGTGGCAGTGGGTCTGGGACACATTTTTCTCTCACTATTAGCAACCTGGAACAAGAAGATATTGGCACTTACTTTTGC	CACCAGGATACTAAGCCTCCGTATACG
**NRC-2895 (HspX)**	**IgG1 (MW861695)**	GACGTGAAGCTGGTGGAGTCTGGGGGAGGTTTAGTGAAGCCTGGAGGGTCCCTGAAACTCTCCTGTGAAGCCTC	GGATTCACTTTCAGTAGCTATACC	ATGTCTTGGGTTCGCCAGACTCCGGAGAAGAGGCTGGAGTGGGTCGCAACC	ATTAGTTTTGGTGGTAGTTACAGC	TACTATCCAGACAGTGTGAAGGGCCGATTCACCATCTCCAGAGACAATGCCAAGAACACCCTGTACCTGCAAATGAGCAGTCTGAAGTCTGAGGACACAGCCATGTATTACTGT	ACAAGAGATCGAGGGGGTAACTACCCGTTTGCTTAC
	**Ig light chain (MW861703)**	CAGGCTGTTGTGACTCAGGAATCTGCACTCACCACATCACCTGGTGAAACAGTCACACTCACTTGTCGCTCAAGT	ACTGGGGCTGTTACAACTAGTAACTAT	GCCAACTGGGTCCAAGAAAAACCAGATCATTTATTCACTGGTCTAATAGGT	GGTACCAAC	AACCGAGCTCCAGGTGTTCCTGCCAGATTCTCAGGCTCCCTGATTGGAGACAAGGCTGCCCTCACCATCACAGGGGCACAGACTGAGGATGAGGCAATATATTTCTGT	GCTCTATGGTACAGCAACCATTGGGTG

**NRC-2897 (Ag85 complex)**	**IgM (MW861696)**	GACGTGAAGCTCGTGGAGTCTGGGGGAGGCTTAGTGAAGCTTGGAGGGTCCCTGAAACTCTCCTGTGCAGCCTCT	GGATTCACTTTCAGTAGCTATTAC	ATGTCTTGGGTTCGCCAGACTCCAGAGAAGAGGCTGGAGTTGGTCGCAGCC	ATTAATAGTAATGGTGGTAGCACC	TACTATCCAGACATTGTGAAGGGCCGATTCACCATCTCCAGAGACAATGCCAAGAACACCCTGTACCTGCAAATGAGCAGTCTGAAGTCTGAGGACACAGCCTTGTATTACTGT	GCAAGACATGGAGGTAACTACCTCGCCTGGTTTGCTTAC
	**Igκ (MW861704)**	GACATTGTGATGACCCAGTCTCAAAAATTCATGTCCACATCAGTAGGAGACAGGGTCAGCGTCACCTGCAAGGCCAGT	CAGAATGTGGGTACTAAT	GTAGCCTGGTATCAACAGAAACCAGGGCAATCTCCTAAAGCACTGATTTAC	TCGGCATCC	TACCGGTACAGTGGAGTCCCTGATCGCTTCACAGGCAGTGGATCTGGGACAGATTTCACTCTCACCATCAGCAATGTGCAGTCTGAAGACTTGGCAGAGTATTTCTGT	CAGCAATATAACAGCTATCCTTACACG
**NRC-2914 (HBHA)**	**IgG2a (MW861697)**	CAGGTCCAACTGCAGCAGCCTGGGGCTGAGTTTGTGAAGCCTGGGACTTCAGTGAAGGTGTCCTGTAAGACTTCT	GGCTACAACTTCACCAGACACTGG	ATAAACTGGGTGAAGCTGAGGCCTGGACAAGGCCTTGAGTGGATTGGAGAT	ATTTATCCTGGTAGTGGTGAGACT	AATTACAATGGGAAGTTCGAAAACAAGGCCACACTGACTGTAGACATATCCTCCAGCACAGCCTACCTGCAACTCAGCAGCCTGGCATCTGAGGACTCTGCTCTCTATTACTGT	GCAAGATATGATTACGACGTTGACTAC
	**Ig light chain (MW861705)**	GACATCAAGATGACCCAGTCTCCATCTTCCATTTATGCATCTCGAGGAGAGAGAGTCACTATCACTTGCAAGGCGAGT	CAGGACATTAATAGCTAT	TTAAGCTGGATCCAGCAGAACCCAGGGAAATCTCCTAAGACCCTGATCTAT	CGTGCAAGC	AGATTGGTAGATGGGGTCCCATCAAGGTTCAGTGGCAGTGGATCTGGGCAAGATTATTCTCTCACCATCAGCAGCCTGGAATATGAAGATATGGGAATTTATTATTGT	CAACAGTATGATGAGTTTCCGCTCACG
**NRC-47679[@] (DnaK)**	**IgG1 (MW861698)**	CAGGTCCACCTTCAGCAGTCTGGGGCTGAACTGGTAAAACCTGGGGCCTCAGTGAAGATGTCCTGCAAGGCTTCT	GGCTACACCTTTACTACCTACTGG	ATGCACTGGGTAAAACAGAGGCCTGGACAGGGTCTGGAATGGATTGGATAC	ATTAATCCTAGCACTGGTTATACT	GAGTACAATCAGAAGTTCAAGGACAAGGCCACATTGACTGCAGACAAATCCTCCAGCACAGCCTACATGGAACTGAGCAGCCTGACATCTGAGGACTCAGCAGTCTATTAC	TGTGCAAGAAACTCCTGGTTTGCTTACTGG
	**Igκ (MW861706)**	GATATTGTGATGACGCAGGCTGCATTCTCCAATCCAGTCACTCTTGGAACATCAGCTTCCATCTCCTGCAGGTCTAGT	AAGAGTCTCCTACATAGTAATGGCATCACTTAT	TTGTATTGGTATCTGCAGAAGCCAGGCCAGTCTCCTCAGCTCCTGATTTAT	CAGATGTCC	AACCTTGCCTCAGGAGTCCCAGACAGGTTCAGTTGCAGTGGGTCAGGAACTGATTTCACACTGAGAATCAGCAGAGTGGAGGCTGAGAATGTGGGTGTTTATTAC	TGTGCTCAAAATCTAGAACTTCCGTGGACGTTC
**NRC-50100[@] (DnaK)**	**IgG1 (MW861699)**	CAGGTCCACCTTCAGCAGTCTGGGGCTGAACTGGTAAAACCTGGGGCCTCAGTGAAGATGTCCTGCAAGGCTTCT	GGCTACACCTTTACTACCTACTGG	ATGCACTGGGTAAAACAGAGGCCTGGACAGGGTCTGGAATGGATTGGATAC	ATTAATCCTAGCACTGGTTATACT	GAGTACAATCAGAAGTTCAAGGACAAGGCCACATTGACTGCAGACAAATCCTCCAGCACAGCCTACATGGAACTGAGCAGCCTGACATCTGAGGACTCAGCAGTCTATTAC	TGTGCAAGAAACTCCTGGTTTGCTTACTGG

	**Igκ (MW861707)**	GATATTGTGATGACGCAGGCTGCATTCTCCAATCCAGTCACTCTTGGAACATCAGCTTCCATCTCCTGCAGGTCTAGT	AAGAGTCTCCTACATAGTAATGGCATCACTTAT	TTGTATTGGTATCTGCAGAAGCCAGGCCAGTCTCCTCAGCTCCTGATTTAT	CAGATGTCC	AACCTTGCCTCAGGAGTCCCAGACAGGTTCAGTTGCAGTGGGTCAGGAACTGATTTCACACTGAGAATCAGCAGAGTGGAGGCTGAGAATGTGGGTGTTTATTAC	TGTGCTCAAAATCTAGAACTTCCGTGGACGTTC
**NRC-50101 (KatG)**	**IgM (MW861700)**	CAGGTGCAGCTGAAGGAGTCAGGACCTGGCCTGGTGGCGCCCTCACAGAGCCTGTCCATCACTTGCACTGTCTCT	GGGTTTTCATTAACCAGCTATGGT	GTACACTGGGTTCGCCAGCCTCCAGGAAAGGGTCTGGAGTGGCTGGGAGTA	ATATGGGCTGGTGGAAGCACA	AATTATAATTCGGCTCTCATGTCCAGACTGAGCATCAGCAAAGACAACTCCAAGAGCCAAGTTTTCTTAAAAATGAACAGTCTGCAAACTGATGACACAGCCATGTACTACTGT	GCCAGAGATGGGTTTGCTTAC
	**Igκ (MW861708)**	GATGTTGTGATGACCCAAACTCCACTCTCCCTGCCTGTCAGTCTTGGAGATCAAGCCTCCATCTCTTGCAGATCTAGT	CAGAGCCTTGTACACAGTAATGGAAACACCTAT	TTACATTGGTACCTGCAGAAGCCAGGCCAGTCTCCAAAGCTCCTGATCTAC	AAAGTTTCC	AACCGATTTTCTGGGGTCCCAGACAGGTTCAGTGGCAGTGGATCAGGGACAGATTTCACACTCAAGATCAGCAGAGTGGAGGCTGAGGATCTGGGAGTTTATTTC	TGCTCTCAAAGTACACATGTTCCTCCGACGTTC
**NRC-50703 (Mpt64)**	**IgG1 (MW861701)**	GACGTGATGCTCGAGGAGTCTGGGGGAGGCTTAGTGAAGCTTGGAGGGTCCCTGAAACTCTCCTGTGCAGCCTCT	GGATTCAGTTTCAGTAGCCATTAC	ATGTCTTGGGTTCGCCAGACTCCGGAGAAGAGGCTGGAGTGGGTCGCAACC	ATTAGTAATGATGGTGGCAGCACC	TACTATCCAGACAGTGTGAAGGACCGATTCACCATCTCCAGAGACAATGCCAAGAACACCCTGTACCTGCAAATGAGTAGTCTGAATTCTGAGGACACAGCCGTGTATTAT	TGTGCAAGGTATAGGTACTTTGATTTTTGG
	**Igκ (MW861709)**	GAAAATGTGCTCACCCAGTCTCCAGCAATCATGTCTGCATCTCTAGGGGAGAAGGTCACCATGAGTTGCAGGGCCAGT	TCAAGTGCAAATTAC	ATGTACTGGTACCAGCAGAAGTCAGATGCCTCCCCCACACTATTGATTTAT	TACACATCC	AACCTGGCTCCTGGAGTCCCAAGTCGCTTCAGTGGCAGTGGGTCTGGGAACTCTTATTCTCTCACAATCAGCAGCATGGAGGGTGAAGATGTTGCCACTTATTAC	TGCCAGCAGTTTACTACTTCCCCATCCATGTACACGTTC
**NRC-2410 (PhoS1/PstS1)**	**IgG1 (MW812375)**	CAGGTTCAGCTGCAGCAGTCTGGAGCTGAACTGATGAAGCCTGGGGCCTCAGTGAAGATATCCTGCAAGGCAACT	GGCTACACATTCAGTGGTTACTGG	GTAGAGTGGGTAAAGCAGAGGCCTGGACATGGCCTTGAGTGGATTGGAGAG	ATTTTACCTGGAAGAGTTAGCACT	AATTACAATGAGAAGTTCAAGGCCAAGGCCACATTCACTGCAGATACATCCTCCAACACAGCCTACATGCAACTCAGCAGCCTGACATCTGAGGACTCTGCCGTCTATTAC	TGTGCAAGATTCAAGAATTACTACGGTAGTAGTTACAACTACTTTGACTACTGG
	**Igκ (MW812376)**	GATATTGTGCTGACTCAGGCTGCACCCTCTGTGCCTGTCACTCCTGGAGAGTCACTTTCCATCTCCTGCAGGTCTAGT	AAGAGTCTCCTGCATAGTAATGGCAACACTTAC	TTGTATTGGTTCCTACAGAGGCCAGGCCAGTCTCCTCAACTCCTGATATAT	CGGATGTCC	AACCTTGCCTCAGGAGTCCCAGACAGGTTCAGTGGCAGTGGGTCAGGAACTGCTTTCACACTGAGAATCAGTAGAGTGGAGGCTGAGGATGTGGGTGTTTATTAC	TGTATGCAACATCTAGAATATCCGTACACGTTC

**NRC-13810 (SodA)**	**IgG1 (MW812377)**	GAAGTGAGGCTTGA<br>GGAGTCTGGAGGA<br>GGCTTGGTGCTACC<br>TGGAGGATCCATGA<br>AACTCTCCTGTGTT<br>GCCTCT	GGATTCACTTTC<br>AATAACTACTGG	ATGAACTGGGTCC<br>GCCAGTCTCCAGA<br>GAAGGGGCTTGA<br>GTGGGTTGCTGAA	ATTAGATTGAA<br>ATCTAATAATT<br>ATGCAACA	CATTATGCGGAGTCTG<br>TGAAAGGGAGGTTCAC<br>CATCTCAAGAGATGATT<br>CCAAAGGTGGTGTCTA<br>CCTGCAAATGAACAAC<br>TTAAGAGCTGAAGACA<br>CTGGCATTTATTAC	TGTACCAGGG<br>AGGCCAACAG<br>GGGGTTTGCT<br>TACTGG
	**Igκ-1 (MW812378)**	AAAATTGTGCTG<br>ACCCAATCTCCA<br>GCTTCTTTGGCT<br>GTGTCTCTAAGG<br>CAGAGGGCCACC<br>ATATCCTGCAGA<br>GCCAGTS	GAAAGTGTTG<br>ATAGTTATGG<br>CAAAAGTTTT	ATGCACTGGTA<br>CCAGCAGAAAT<br>CAGGACAGCCA<br>CCCAAACTCCTC<br>ATCTAT	CGTGCATCC	AACCTAGAATCTGG<br>GGTCCCTGCCAGGT<br>TCAGTGGCAGTGGG<br>TCTAGGACAGACTT<br>CACCCTCACCATTG<br>ATCCTGTGGAGGCT<br>GATGATGCTGCAAC<br>CTATTACTGT	CAGCAAAAT<br>TATGAGGCT<br>CCTCGGACG
	**Igκ-2 (MW812379)**	GACATTGTGCTG<br>ACACAGTCTCCT<br>GCTTCTTTGGCT<br>GTGTCTCTAAGG<br>CAGAGGGCCACC<br>ATATCCTGCAGA<br>GCCAGTS	GAAAGTGTTG<br>ATAGTTATGG<br>CAAAAGTTTT	ATGCACTGGTA<br>CCAGCAGAAAT<br>CAGGACAGCCA<br>CCCAAACTCCTC<br>ATCTAT	CGTGCATCC	AACCTAGAATCTGG<br>GGTCCCTGCCAGGT<br>TCAGTGGCAGTGGG<br>TCTAGGACAGACTT<br>CACCCTCACCATTG<br>ATCCTGTGGAGGCT<br>GATGATGCTGCAAC<br>CTATTACTGT	CAGCAAAAT<br>TATGAGGCT<br>CCTCGGACG
**NRC-49680 (KatG)**	**IgM (MW812380)**	CAGGTGCAGCTG<br>AAGGAGTCAGGA<br>CCTGGCCTGGTG<br>GCGCCCTCACAG<br>AGCCTGTCCATC<br>ACATGCACTGTC<br>TCA	GGGTTCTCAT<br>TAACCGACTA<br>TGGT	GTAAGCTGGATT<br>CGCCAGCCTCC<br>AGGAAAGGGTC<br>TGGAGTGGCTG<br>GGAGTA	ATATGGGGT<br>GGTGGAAGC<br>ACA	TACTATAATTCAGCT<br>CTCAAATCCAGACT<br>GAGCATCAGCAAGG<br>ACAACTCCAAGAGC<br>CAAGTTTTCTTAAAA<br>ATGAACAGTCTGCA<br>AACTGATGACACAG<br>CCATGTACTACTGT	GCCAAACAT<br>GGTAACTTT<br>GCTTAC
	**Igκ (MW812381)**	CAAATTGTTCTCA<br>CCCAGTCTCCAG<br>CAATCATGTCTG<br>CATCTCTAGGGG<br>AACGGGTCACCA<br>TGACCTGCACTG<br>CCAGC	TCAAGTGTAA<br>GTTCCAGTTA<br>C	TTGCACTGGTAC<br>CAGCAGAAGCC<br>AGGATCCTCCC<br>CCAAACTCTGG<br>ATTTAT	AGCACATCC	AACCTGGCTTCTGG<br>AGTCCCAGCTCGCT<br>TCAGTGGCAGTGGG<br>TCTGGGACCTCTTA<br>CTCTCTCACAATCAG<br>CAGCATGGAGGCTG<br>AAGATGCTGCCACT<br>TATTACTGC	CACCAGTAT<br>CATCGTTCC<br>CCGTGGACG
**NRC-2894 (GroES)**	**IgG2a (MW812373)**	GAGGTGCAGCTT<br>GTTGAGTCTGGT<br>GGAGGATTGGTG<br>CAGCCTAAAGGG<br>TCATTGAAACTCT	GGATTCACCT<br>TCAAAACCTA<br>CGCC	ATGAACTGGGT<br>CCGCCACACTC<br>CAGGAAAGGGT<br>TTGGAATGGGTT<br>GCTCGC	ATAAGAAGTA<br>AAAGTAATAA<br>TTTTGCAACA	TATTATGCCGATTCA<br>GTGAAAGACCGGTT<br>CACCATCTCCAGAG<br>ATGATTCACAAAGCA<br>TGCTCTATCTGCAAA<br>TGAACAACTTGAAAA	GTGAAACTA<br>ACTAACGGC<br>TACTTTGACT<br>CC

		CATGTGCAGCCT CT				CTGAGGACACAGCC ATGTATTACTGT	
	**Igκ (MW812374)**	GACATCCAGATG ACACAGTCTCCA TCCTCACTGTCT GCATCTCTGGGA GGCAAAGTCACC ATCACTTGCAAG GCAAGC	CAAGACATTA ACAATTAT	ATAGCTTGGTAC CAACACAAGCC TGGAAAAGGTC CTAGGCTGCTC ATACAT	GACACATCA	ACATTACAGCCAGG CATCCCATCAAGGTT CAGTGGAAGTGGGT CTGGGAGAGATTAT TCCTTCAGCATCAG CAACCTGGAGCCTG AAGATATTGCAACTT ATTATTGT	CTACAGTAT GATAATCTTC GGACG

Symbols (@) indicate the same Ig composite between clones
