# Supplementary material for: Challenges and Potential of Antibody–Drug Conjugates as Prospective Tuberculosis Therapeutics
Source: Microorganisms. 2025 Sep 24;13(10):2234. doi: 10.3390/microorganisms13102234 (PMC12566324; doi:10.3390/microorganisms13102234)
Supplement: Supplementary file 1 [file microorganisms-13-02234-s001.zip › Foreman_MtbADC_KABAT_Table S2.pdf]

Table S2. Summary of the nucleic acid sequences of immunoglobulin light and heavy chains based on the KABAT definition

Hybridoma	Ig (Genbank #)	FR1	CDR1	FR2	CDR2	FR3	CDR3	FR4
**NRC-13806 (LprG/p27)**	**IgG1 (MW854314)**	CAGGTCCAACTGCAGCAGTCTGGGGCTGAACTGGTGAAGCCTGGGGCTTCAGTGAAGTTGTCCTGCAAGGCTTCTGGCTACACCTTCACC	AACTACTATATGTAC	TGGGTGAGGCAGAGGCCTGGACAAGGCCTTGAGTGGATTGGA	GAGATTTATCCTAGCAATGGTGGTACTAACTTCAATGAGAAGTTCAAGAAC	AAGGCCACACTGACTGTAGACAAATCTTCCACCACAGCATACATGCAGCTCAGCAGCCTGACATCTGAAGACTCTGCGATCTATTACTGTACAACT	CACCTTGCTTAC	TGGGGCCAAGGGACTCTGGTCACTGTCTCTGCA
	**Igκ (MW854315)**	GATGTTTTGATGACCCAAACTCCACTCTCCCTGCCTGTCAGTCTTGGAGATCAAGCCTCCATCTCTTGC	AGATCTAGTCAGAGCATTGTACATAGTAATGGAAACACCTATTTAGAA	TGGTACCTGCAGAAACCAGGCCAGTCTCCAAACCTCCTGATCTAC	AAAGTTTCCAACCGATTTTCT	GGGGTCCCAGACAGGTTCAGTGGCAGTGGATCAGGGACAGATTTCACACTCAAGATCAGCAGAGTGGAGGCTGAGGATCTGGGAGTTTATTACTGC	TTTCAAGGTTCACATGCTCCGTACACG	TTCGGAGGGGGGACCAAGCTGGAAATAAAA
**NRC-2893 (LAM)**	**IgG3 (MW854316)**	GAGGTTCAGCTCCAGCAGTCTGGGACTGTGCTGGCAAGGCCTGGGACTTCCGTGAAGATGTCCTGCAAGGCTTCTGGCTACAGCTTTACC	AACTACTGGATGCAC	TGGGTAAAACAGAGGCCTGGACAGGGTCTAGAGTGGATTGGT	TCTATTTATCCTGGAAATAGTGATACTAACTACAAGCAGAAATTCAAGGGC	AAGGCCAAACTGACTGCAGTCACATCCGCCAGCACTGCCTACATGGAGGTCAACAGCCTGACAAATGAGGACTCTGCGGTCTATTACTGTACAAGA	TTTGGTAACTACGTTCCGTTTGCTTAC	TGGGGCCAAGGGACTCTGGTCACTGTCTCTGCA
	**Igκ (MW854317)**	GATATCCAGATGACACAGACTACATCCTCCCTGTCTGCCTCTCTGGGAGACAGAGTCACCATCGGTTGC	AGGGCAAGTCAGGACATTGGCAGTTATTTAAAC	TGGTATCAGCAGAAACCAGATGGAGCTGTTAGACTCCTGATCTAC	TACACATCAAGATTACACTCA	GGAGTCCCATCAAGGTTCAGTGGCAGTGGGTCTGGGACACATTTTTCTCTCACTATTAGCAACCTGGAACAAGAAGATATTGGCACTTACTTTTGC	CACCAGGATACTAAGCCTCCGTATACG	TTCGGATCGGGGACCAAGCTGGAAATAAAA
**NRC-2895 (HspX)**	**IgG1 (MW861695)**	GACGTGAAGCTGGTGGAGTCTGGGGGAGGTTTAGTGAAGCCTGGAGGGTCCCTGAAACTCTCCTGTGAAGCCTCTGGATTCACTTTCAGT	AGCTATACCATGTCT	TGGGTTCGCCAGACTCCGGAGAAGAGGCTGGAGTGGGTCGCA	ACCATTAGTTTTGGTGGTAGTTACAGCTACTATCCAGACAGTGTGAAGGGC	CGATTCACCATCTCCAGAGACAATGCCAAGAACACCCTGTACCTGCAAATGAGCAGTCTGAAGTCTGAGGACACAGCCATGTATTACTGTACAAGA	GATCGAGGGGGTAACTACCCGTTTGCTTAC	TGGGGCCAAGGGACTCTGGTCACTGTCTCTGCA
	**Ig light chain (MW861703)**	CAGGCTGTTGTGACTCAGGAATCTGCACTCACCACATCACCTGGTGAAACAGTCACACTCACTTGT	CGCTCAAGTACTGGGGCTGTTACAACTAGTAACTATGCCAAC	TGGGTCCAAGAAAAACCAGATCATTTATTCACTGGTCTAATAGGT	GGTACCAACAACCGAGCTCCA	GGTGTTCCTGCCAGATTCTCAGGCTCCCTGATTGGAGACAAGGCTGCCCTCACCATCACAGGGGCACAGACTGAGGATGAGGCAATATATTTCTGT	GCTCTATGGTACAGCAACCATTGGGTG	TTCGGTGGAGGAACCAAACTGACTGTCCTA
**NRC-2897**	**IgM (MW861696)**	GACGTGAAGCTCGTGGAGTCTGGGGGAGGCTTAGTGAAGCTTGGAGGGTCCCTGAAACTCTCCTGTGCAGC	AGCTATTACATGTCT	TGGGTTCGCCAGACTCCAGAGAAGAGGCTGGAGTTGGTCGCA	GCCATTAATAGTAATGGTGGTAGCACCTACTATCCAGACA	CGATTCACCATCTCCAGAGACAATGCCAAGAACACCCTGTACCTGCAAATGAGCAGTCTGAAGTCTGAGGAC	CATGGAGGTAACTACCTCGCCTGGTTTGCTTAC	TGGGGCCAAGGGACTCTGGTCACTGTCTCTGCA

**(Ag85 complex)**		CTCTGGATTCACTTTCAGT			TTGTGAAGGGC	ACAGCCTTGTATTACTGTGCAAGA		
	**Igκ (MW861704)**	GACATTGTGATGACCCAGTCTCAAAAATTCATGTCCACATCAGTAGGAGACAGGGTCAGCGTCACCTGC	AAGGCCAGTCAGAATGTGGGTACTAATGTAGCC	TGGTATCAACAGAAACCAGGGCAATCTCCTAAAGCACTGATTTAC	TCGGCATCCTACCGGTACAGT	GGAGTCCCTGATCGCTTCACAGGCAGTGGATCTGGGACAGATTTCACTCTCACCATCAGCAATGTGCAGTCTGAAGACTTGGCAGAGTATTTCTGT	CAGCAATATAACAGCTATCCTTACACG	TTCGGAGGGGGGACCAAGCTGGAAATAAAA
**NRC-2914 (HBHA)**	**IgG2a (MW861697)**	CAGGTCCAACTGCAGCAGCCTGGGGCTGAGTTTGTGAAGCCTGGGACTTCAGTGAAGGTGTCCTGTAAGACTTCTGGCTACAACTTCACC	AGACACTGGATAAAC	TGGGTGAAGCTGAGGCCTGGACAAGGCCTTGAGTGGATTGGA	GATATTTATCCTGGTAGTGGTGAGACTAATTACAATGGGAAGTTCGAAAAC	AAGGCCACACTGACTGTAGACATATCCTCCAGCACAGCCTACCTGCAACTCAGCAGCCTGGCATCTGAGGACTCTGCTCTCTATTACTGTGCAAGA	TATGATTACGACGTTGACTAC	TGGGGCCAAGGCACCGCTCTCACAGTCTCCTCA
	**Ig light chain (MW861705)**	GACATCAAGATGACCCAGTCTCCATCTTCCATTTATGCATCTCGAGGAGAGAGAGTCACTATCACTTGC	AAGGCGAGTCAGGACATTAATAGCTATTTAAGC	TGGATCCAGCAGAACCCAGGGAAATCTCCTAAGACCCTGATCTAT	CGTGCAAGCAGATTGGTAGAT	GGGGTCCCATCAAGGTTCAGTGGCAGTGGATCTGGGCAAGATTATTCTCTCACCATCAGCAGCCTGGAATATGAAGATATGGGAATTTATTATTGT	CAACAGTATGATGAGTTTCCGCTCACG	TTCGGTGGTGGGACCAAGCTGGAGCTGAAA
**NRC-47679@ (DnaK)**	**IgG1 (MW861698)**	CAGGTCCACCTTCAGCAGTCTGGGGCTGAACTGGTAAAACCTGGGGCCTCAGTGAAGATGTCCTGCAAGGCTTCTGGCTACACCTTTACT	ACCTACTGGATGCAC	TGGGTAAAACAGAGGCCTGGACAGGGTCTGGAATGGATTGGA	TACATTAATCCTAGCACTGGTTATACTGAGTACAATCAGAAGTTCAAGGAC	AAGGCCACATTGACTGCAGACAAATCCTCCAGCACAGCCTACATGGAACTGAGCAGCCTGACATCTGAGGACTCAGCAGTCTATTACTGTGCAAGA	AACTCCTGGTTTGCTTAC	TGGGGCCAAGGGACTCTGGTCACTGTCTCTGCA
	**Igκ (MW861706)**	GATATTGTGATGACGCAGGCTGCATTCTCCAATCCAGTCACTCTTGGAACATCAGCTTCCATCTCCTGC	AGGTCTAGTAAGAGTCTCCTACATAGTAATGGCATCACTTATTTGTAT	TGGTATCTGCAGAAGCCAGGCCAGTCTCCTCAGCTCCTGATTTAT	CAGATGTCCAACCTTGCCTCA	GGAGTCCCAGACAGGTTCAGTTGCAGTGGGTCAGGAACTGATTTCACACTGAGAATCAGCAGAGTGGAGGCTGAGAATGTGGGTGTTTATTACTGT	GCTCAAAATCTAGAACTTCCGTGGACG	TTCGGTGGAGGCACCAAGCTGGAAATCAAA
**NRC-50100@ (DnaK)**	**IgG1 (MW861699)**	CAGGTCCACCTTCAGCAGTCTGGGGCTGAACTGGTAAAACCTGGGGCCTCAGTGAAGATGTCCTGCAAGGCTTCTGGCTACACCTTTACT	ACCTACTGGATGCAC	TGGGTAAAACAGAGGCCTGGACAGGGTCTGGAATGGATTGGA	TACATTAATCCTAGCACTGGTTATACTGAGTACAATCAGAAGTTCAAGGAC	AAGGCCACATTGACTGCAGACAAATCCTCCAGCACAGCCTACATGGAACTGAGCAGCCTGACATCTGAGGACTCAGCAGTCTATTACTGTGCAAGA	AACTCCTGGTTTGCTTAC	TGGGGCCAAGGGACTCTGGTCACTGTCTCTGCA
	**Igκ (MW861707)**	GATATTGTGATGACGCAGGCTGCATTCTCCAATCCAGTCACTCTTGGAACATCAGCTTCCATCTCCTGC	AGGTCTAGTAAGAGTCTCCTACATAGTAATGGCATCACTTATTTGTAT	TGGTATCTGCAGAAGCCAGGCCAGTCTCCTCAGCTCCTGATTTAT	CAGATGTCCAACCTTGCCTCA	GGAGTCCCAGACAGGTTCAGTTGCAGTGGGTCAGGAACTGATTTCACACTGAGAATCAGCAGAGTGGAGGCTGAGAATGTGGGTGTTTATTACTGT	GCTCAAAATCTAGAACTTCCGTGGACG	TTCGGTGGAGGCACCAAGCTGGAAATCAAA
**NRC-50101 (KatG)**	**IgM (MW861700)**	CAGGTGCAGCTGAAGGAGTCAGGACCTGGCCTGGTGGCGCCC	AGCTATGGTGTACAC	TGGGTTCGCCAGCCTCCAGGAAAGGG	GTAATATGGGCTGGTGGAAGCACA	AGACTGAGCATCAGCAAAGACAACTCCAAGAGCCAAGTTTTCTTAAAAATGAACA	GATGGGTTTGCTTAC	TGGGGCCAAGGGACTCTGGT

		TCACAGAGCCTGTCCATCACTTGCACTGTCTCTGGGTTTTCATTAACC		TCTGGAGTGGCTGGGA	AATTATAATTCGGCTCTCATGTCC	GTCTGCAAACTGATGACACAGCCATGTACTACTGTGCCAGA		CACTGTCTCTGCA
	**Igκ (MW861708)**	GATGTTGTGATGACCCAAACTCCACTCTCCCTGCCTGTCAGTCTTGGAGATCAAGCCTCCATCTCTTGC	AGATCTAGTCAGAGCCTTGTACACAGTAATGGAAACACCTATTTACAT	TGGTACCTGCAGAAGCCAGGCCAGTCTCCAAAGCTCCTGATCTAC	AAAGTTTCCAACCGATTTTCT	GGGGTCCCAGACAGGTTCAGTGGCAGTGGATCAGGGACAGATTTCACACTCAAGATCAGCAGAGTGGAGGCTGAGGATCTGGGAGTTTATTTCTGC	TCTCAAAGTACACATGTTCCTCCGACG	TTCGGTGGAGGCACCAAGCTGGAAATCAAA
**NRC-50703 (Mpt64)**	**IgG1 (MW861701)**	GACGTGATGCTCGAGGAGTCTGGGGGAGGCTTAGTGAAGCTTGGAGGGTCCCTGAAACTCTCCTGTGCAGCCTCTGGATTCAGTTTCAGT	AGCCATTACATGTCT	TGGGTTCGCCAGACTCCGGAGAAGAGGCTGGAGTGGGTCGCA	ACCATTAGTAATGATGGTGGCAGCACCTACTATCCAGACAGTGTGAAGGAC	CGATTCACCATCTCCAGAGACAATGCCAAGAACACCCTGTACCTGCAAATGAGTAGTCTGAATTCTGAGGACACAGCCGTGTATTATTGTGCAAGG	TATAGGTACTTTGATTTT	TGGGGCCAAGGCACCACTCTCACAGTCTCCTCA
	**Igκ (MW861709)**	GAAAATGTGCTCACCCAGTCTCCAGCAATCATGTCTGCATCTCTAGGGGAGAAGGTCACCATGAGTTGC	AGGGCCAGTTCAAGTGCAAATTACATGTAC	TGGTACCAGCAGAAGTCAGATGCCTCCCCCACACTATTGATTTAT	TACACATCCAACCTGGCTCCT	GGAGTCCCAAGTCGCTTCAGTGGCAGTGGGTCTGGGAACTCTTATTCTCTCACAATCAGCAGCATGGAGGGTGAAGATGTTGCCACTTATTACTGC	CAGCAGTTTACTACTTCCCCATCCATGTACACG	TTCGGAGGGGGGACCAAGCTGGAAATAAAA
**NRC-2410 (PhoS1/PstS1)**	**IgG1 (MW812375)**	CAGGTTCAGCTGCAGCAGTCTGGAGCTGAACTGATGAAGCCTGGGGCCTCAGTGAAGATATCCTGCAAGGCAACTGGCTACACATTCAGT	GGTTACTGGGTAGAG	TGGGTAAAGCAGAGGCCTGGACATGGCCTTGAGTGGATTGGA	GAGATTTTACCTGGAAGAGTTAGCACTAATTACAATGAGAAGTTCAAGGCC	AAGGCCACATTCACTGCAGATACATCCTCCAACACAGCCTACATGCAACTCAGCAGCCTGACATCTGAGGACTCTGCCGTCTATTACTGTGCAAGA	TTCAAGAATTACTACGGTAGTAGTTACAACTACTTTGACTAC	TGGGGCCAAGGCACCACTCTCACAGTTTCCTCA
	**Igκ (MW812376)**	GATATTGTGCTGACTCAGGCTGCACCCTCTGTGCCTGTCACTCCTGGAGAGTCACTTTCCATCTCCTGC	AGGTCTAGTAAGAGTCTCCTGCATAGTAATGGCAACACTTACTTGTAT	TGGTTCCTACAGAGGCCAGGCCAGTCTCCTCAACTCCTGATATAT	CGGATGTCCAACCTTGCCTCA	GGAGTCCCAGACAGGTTCAGTGGCAGTGGGTCAGGAACTGCTTTCACACTGAGAATCAGTAGAGTGGAGGCTGAGGATGTGGGTGTTTATTACTGT	ATGCAACATCTAGAATATCCGTACACG	TTCGGAGGGGGGACCAAGCTGGAAATAAAA
**NRC-13810 (SodA)**	**IgG1 (MW812377)**	GAAGTGAGGCTTGAGGAGTCTGGAGGAGGCTTGGTGCTACCTGGAGGATCCATGAAACTCTCCTGTGTTGCCTCTGGATTCACTTTCAAT	AACTACTGGATGAAC	TGGGTCCGCCAGTCTCCAGAGAAGGGGCTTGAGTGGGTTGCT	GAAATTAGATTGAAATCTAATAATTATGCAACACATTATGCGGAGTCTGTGAAAGGG	AGGTTCACCATCTCAAGAGATGATTCCAAAGGTGGTGTCTACCTGCAAATGAACAACTTAAGAGCTGAAGACACTGGCATTTATTACTGTACCAGG	GAGGCCAACAGGGGGTTTGCTTAC	TGGGGCCAAGGGACTCTGGTCACTGTCTCTGCA
	**Igκ-1 (MW812378)**	AAAATTGTGCTGACCCAATCTCCAGCTTCTTTGGCTGTGTCTCTAAGGCAGAGGGCCACCATATCCTGC	AGAGCCAGTGAAAGTGTTGATAGTTATGGCAAAAGTTTTATGCAC	TGGTACCAGCAGAAATCAGGACAGCCACCCAAACTCCTCATCTAT	CGTGCATCCAACCTAGAATCT	GGGGTCCCTGCCAGGTTCAGTGGCAGTGGGTCTAGGACAGACTTCACCCTCACCATTGATCCTGTGGAGGCTGATGATGCTGCAACCTATTACTGT	CAGCAAAATTATGAGGCTCCTCGGACG	TTCGGTGGAGGCACCAAGCTGGAAATCAAA

	**Igκ-2 (MW812379)**	GACATTGTGCTGACACAGTCTCCTGCTTCTTTGGCTGTGTCTCTAAGGCAGAGGGCCACCATATCCTGC	AGAGCCAGTGAAAGTGTTGATAGTTATGGCAAAAGTTTTATGCAC	TGGTACCAGCAGAAATCAGGACAGCCACCCAAACTCCTCATCTAT	CGTGCATCCAACCTAGAATCT	GGGGTCCCTGCCAGGTTCAGTGGCAGTGGGTCTAGGACAGACTTCACCCTCACCATTGATCCTGTGGAGGCTGATGATGCTGCAACCTATTACTGT	CAGCAAAATTATGAGGCTCCTCGGACG	TTCGGTGGAGGCACCAAGCTGGAAATCAAA
**NRC-49680 (KatG)**	**IgM (MW812380)**	CAGGTGCAGCTGAAGGAGTCAGGACCTGGCCTGGTGGCGCCCTCACAGAGCCTGTCCATCACATGCACTGTCTCAGGGTTCTCATTAACC	GACTATGGTGTAAGC	TGGATTCGCCAGCCTCCAGGAAAGGGTCTGGAGTGGCTGGGA	GTAATATGGGGTGGTGGAAGCACATACTATAATTCAGCTCTCAAATCC	AGACTGAGCATCAGCAAGGACAACTCCAAGAGCCAAGTTTTCTTAAAAATGAACAGTCTGCAAACTGATGACACAGCCATGTACTACTGTGCCAAA	CATGGTAACTTTGCTTAC	TGGGGCCAAGGGACTCTGGTCACTGTCTCTGCA
	**Igκ (MW812381)**	CAAATTGTTCTCACCCAGTCTCCAGCAATCATGTCTGCATCTCTAGGGGAACGGGTCACCATGACCTGC	ACTGCCAGCTCAAGTGTAAGTTCCAGTTACTTGCAC	TGGTACCAGCAGAAGCCAGGATCCTCCCCCAAACTCTGGATTTAT	AGCACATCCAACCTGGCTTCT	GGAGTCCCAGCTCGCTTCAGTGGCAGTGGGTCTGGGACCTCTTACTCTCTCACAATCAGCAGCATGGAGGCTGAAGATGCTGCCACTTATTACTGC	CACCAGTATCATCGTTCCCCGTGGACG	TTCGGTGGAGGCACCAAGCTGGAAATCAAA
**NRC-2894 (GroES)**	**IgG2a (MW812373)**	GAGGTGCAGCTTGTTGAGTCTGGTGGAGGATTGGTGCAGCCTAAAGGGTCATTGAAACTCTCATGTGCAGCCTCTGGATTCACCTTCAAA	ACCTACGCCATGAAC	TGGGTCCGCCACACTCCAGGAAAGGGTTTGGAATGGGTTGCT	CGCATAAGAAGTAAAAGTAATAATTTTGCAACATATTATGCCGATTCAGTGAAAGAC	CGGTTCACCATCTCCAGAGATGATTCACAAAGCATGCTCTATCTGCAAATGAACAACTTGAAAACTGAGGACACAGCCATGTATTACTGTGTGAAA	CTAACTAACGGCTACTTTGACTCC	TGGGGCCAAGGCACCACTCTCACAGTCTCCTCA
	**Igκ (MW812374)**	GACATCCAGATGACACAGTCTCCATCCTCACTGTCTGCATCTCTGGGAGGCAAAGTCACCATCACTTGC	AAGGCAAGCCAAGACATTAACAATTATATAGCT	TGGTACCAACACAAGCCTGGAAAAGGTCCTAGGCTGCTCATACAT	GACACATCAACATTACAGCCA	GGCATCCCATCAAGGTTCAGTGGAAGTGGGTCTGGGAGAGATTATTCCTTCAGCATCAGCAACCTGGAGCCTGAAGATATTGCAACTTATTATTGT	CTACAGTATGATAATCTTCGGACG	TTCGGTGGAGGCACCAAGGTGGAAATCAAA

Symbols (@) indicate the same Ig composite between clones
