# Supplementary material for: Challenges and Potential of Antibody–Drug Conjugates as Prospective Tuberculosis Therapeutics
Source: Microorganisms. 2025 Sep 24;13(10):2234. doi: 10.3390/microorganisms13102234 (PMC12566324; doi:10.3390/microorganisms13102234)
Supplement: Supplementary file 1 [file microorganisms-13-02234-s001.zip › Foreman_MtbADC_Supplementary S1.pdf]

Supplementary S1: FASTA IgV (Fab) sequences for BEI-Mtb Hybridoma clones in Table 1

A. NRC-13806 (LprG/p27)

>NRC-13806-IgG1, GenBank accession number# MW854314, length:1-598 bps, CDS: 60-598

ATGGGGAAAACATGAGATCACAGTTCTCTCTACAGTTACTGAGCACACAGGAACTCACCATGGGATGGAGCTATATCATCCTCTTTTTGGTA
GCAACAGCAACAGGTGTCCACTCCCAGGTCCAACTGCAGCAGTCTGGGGCTGAACTGGTGAAGCCTGGGGCTTCAGTGAAGTTGTCCTG
CAAGGCTTCTGGCTACACCTTCACCAACTACTATATGTACTGGGTGAGGCAGAGGCCTGGACAAGGCCTTGAGTGGATTGGAGAGATTTAT
CCTAGCAATGGTGGTACTAACTTCAATGAGAAGTTCAAGAACAAGGCCACACTGACTGTAGACAAATCTTCCACCACAGCATACATGCAGCT
CAGCAGCCTGACATCTGAAGACTCTGCGATCTATTACTGTACAACTCACCTTGCTTACTGGGGCCAAGGGACTCTGGTCACTGTCTCTGCA
GCCAAAACGACACCCCCATCTGTCTATCCACTGGCCCCTGGATCTGCTGCCCAAACTAACTCCATGGTGACCCTGGGATGCCTGGTCAAG
GGCTATTTCCCTGAGCCAGTGACAGTGACCTGGAACTCTGGATCCCTGTCCAG

>NRC-13806-Igκ, GenBank accession number# MW854315, length: 1- 466 bps, CDS: 52-466

ATGGGGACTGATCAGTCTCCTCAGGCTGTCTCCTCAGGTTGCCTCCTCAAAATGAAGTTGCCTGTTAGGCTGTTGGTGCTGATGTTCTGGA
TTCCTGCTTCCAGCAGTGATGTTTTGATGACCCAAACTCCACTCTCCCTGCCTGTCAGTCTTGGAGATCAAGCCTCCATCTCTTGCAGATCT
AGTCAGAGCATTGTACATAGTAATGGAAACACCTATTTAGAATGGTACCTGCAGAAACCAGGCCAGTCTCCAAACCTCCTGATCTACAAAGTT
TCCAACCGATTTTCTGGGGTCCCAGACAGGTTCAGTGGCAGTGGATCAGGGACAGATTTCACACTCAAGATCAGCAGAGTGGAGGCTGAG
GATCTGGGAGTTTATTACTGCTTTCAAGGTTCACATGCTCCGTACACGTTCGGAGGGGGGACCAAGCTGGAAATAAAACGGGCTGATGCTG
CACCAACTG

B. NRC-2893 (LAM)

>NRC-2893- IgG3, GenBank accession number# MW854316, length: 1-619 bps, CDS: 35-619

ATGGGGACAGTCATTGAAAACACTGACTCTAATCATGGAATGTAACTGGATACTTCCTTTTATTCTGTCGGTAATTTCAGGGGTCTACTCAGA
GGTTCAGCTCCAGCAGTCTGGGACTGTGCTGGCAAGGCCTGGGACTTCCGTGAAGATGTCCTGCAAGGCTTCTGGCTACAGCTTTACCAA
CTACTGGATGCACTGGGTAAAACAGAGGCCTGGACAGGGTCTAGAGTGGATTGGTTCTATTTATCCTGGAAATAGTGATACTAACTACAAGC
AGAAATTCAAGGGCAAGGCCAAACTGACTGCAGTCACATCCGCCAGCACTGCCTACATGGAGGTCAACAGCCTGACAAATGAGGACTCTG
CGGTCTATTACTGTACAAGATTTGGTAACTACGTTCCGTTTGCTTACTGGGGCCAAGGGACTCTGGTCACTGTCTCTGCAGCTACAACAACA
GCCCCATCTGTCTATCCCTTGGTCCCTGGCTGCAGTGACACATCTGGATCCTCGGTGACACTGGGATGCCTTGTCAAAGGCTACTTCCCTG
AGCCGGTAACTGTAAAATGGAACTATGGAGCCCTGTCCAGCGGTGTGCGCACAGTCTCATCTGTCCTGCAG

>NRC-2893- Igκ, GenBank accession number# MW854317, length: 1-403 bps, CDS 1-403

ATGATGTCCTCTGCTCAGTTCCTTGGTCTCCTGTTGCTCTGTTTTCAAGGTACCAGATGTGATATCCAGATGACACAGACTACATCCTCCCTG
TCTGCCTCTCTGGGAGACAGAGTCACCATCGGTTGCAGGGCAAGTCAGGACATTGGCAGTTATTTAAACTGGTATCAGCAGAAACCAGATG
GAGCTGTTAGACTCCTGATCTACTACACATCAAGATTACACTCAGGAGTCCCATCAAGGTTCAGTGGCAGTGGGTCTGGGACACATTTTTCT
CTCACTATTAGCAACCTGGAACAAGAAGATATTGGCACTTACTTTTGCCACCAGGATACTAAGCCTCCGTATACGTTCGGATCGGGGACCAA
GCTGGAAATAAAACGGGCTGATGCTGCACCAACTG

C. NRC-2895 (HspX)

>NRC-2895- IgG1, GenBank accession number# MW861695, length: 1-667 bps, CDS 90-667

ATGGGGAGCTCTGACAGAGGAGGCCAGTCCTGGAATTGATTCCCAGTTCCTCACGTTCAGTGATGAGCAGTGAACACAGACCCCTCACCA
TGAACTTCGGGCTCAGATTGATTTTCCTTGTCCTTACTTTAAAAGGTGTCCAGTGTGACGTGAAGCTGGTGGAGTCTGGGGGAGGTTTAGT
GAAGCCTGGAGGGTCCCTGAAACTCTCCTGTGAAGCCTCTGGATTCACTTTCAGTAGCTATACCATGTCTTGGGTTCGCCAGACTCCGGAG
AAGAGGCTGGAGTGGGTCGCAACCATTAGTTTTGGTGGTAGTTACAGCTACTATCCAGACAGTGTGAAGGGCCGATTCACCATCTCCAGAG
ACAATGCCAAGAACACCCTGTACCTGCAAATGAGCAGTCTGAAGTCTGAGGACACAGCCATGTATTACTGTACAAGAGATCGAGGGGGTAA
CTACCCGTTTGCTTACTGGGGCCAAGGGACTCTGGTCACTGTCTCTGCAGCCAAAACGACACCCCCATCTGTCTATCCACTGGCCCCTGG
ATCTGCTGCCCAAACTAACTCCATGGTGACCCTGGGATGCCTGGTCAAGGGCTATTTCCCTGAGCCAGTGACAGTGACCTGGAACTCTGG
ATCCCTGTCCAGCGGTGTGCACACCTTCCCAGC

>NRC-2895- Ig light chain, GenBank accession number# MW861703, length: 1-479 bps, CDS 44-479

ATGGGGGACCAATATTGAAAAGAATAGACCTGGTTTGTGAATTATGGCCTGGATTTCACTTATACTCTCTCTCCTGGCTCTCAGCTCAGGGG
CCATTTCCCAGGCTGTTGTGACTCAGGAATCTGCACTCACCACATCACCTGGTGAAACAGTCACACTCACTTGTCGCTCAAGTACTGGGGC
TGTTACAACTAGTAACTATGCCAACTGGGTCCAAGAAAAACCAGATCATTTATTCACTGGTCTAATAGGTGGTACCAACAACCGAGCTCCAG
GTGTTCCTGCCAGATTCTCAGGCTCCCTGATTGGAGACAAGGCTGCCCTCACCATCACAGGGGCACAGACTGAGGATGAGGCAATATATTT
CTGTGCTCTATGGTACAGCAACCATTGGGTGTTCGGTGGAGGAACCAAACTGACTGTCCTAGGCCAGCCCAAGTCTTCGCCATCAGTCAC
CCTGTTTCCACCTTCCTCTGAAG

D. NRC-2897 (Ag85 complex)

>NRC-2897- IgM, GenBank accession number# MW861696, length: 1-623 bps, CDS 87-623

ATGGGGTCTGACAGAGGAGGCCTGTCCTGGATTCGATTCCCAGTTCCTCACATTCAGTGATCAGCACTGAACACAGACCCCTCACCATGAA
CTTCGGGCTCAGATTGATTTTCCTTGTCCTTGTTTTAAAAGGTGTCCTGTGTGACGTGAAGCTCGTGGAGTCTGGGGGAGGCTTAGTGAAG
CTTGGAGGGTCCCTGAAACTCTCCTGTGCAGCCTCTGGATTCACTTTCAGTAGCTATTACATGTCTTGGGTTCGCCAGACTCCAGAGAAGAG
GCTGGAGTTGGTCGCAGCCATTAATAGTAATGGTGGTAGCACCTACTATCCAGACATTGTGAAGGGCCGATTCACCATCTCCAGAGACAATG
CCAAGAACACCCTGTACCTGCAAATGAGCAGTCTGAAGTCTGAGGACACAGCCTTGTATTACTGTGCAAGACATGGAGGTAACTACCTCGCC
TGGTTTGCTTACTGGGGCCAAGGGACTCTGGTCACTGTCTCTGCAGAGAGTCAGTCCTTCCCAAATGTCTTCCCCCTCGTCTCCTGCGAGA
GCCCCCTGTCTGATAAGAATCTGGTGGCCATGGGCTGCCTGGCCCGGGACTTCCTGCCCAGCACCATTTCCTTC

>NRC-2897- Igκ, GenBank accession number# MW861704, length: 1-438 bps, CDS 67-438

ATGGGGGAAATACATCAGATCAGCATGGGCATCAAGATGGAGTCACAGACTCAGGTCTTTGTATACATGTTGCTGTGGTTGTCTGGTGTTGAT
GGAGACATTGTGATGACCCAGTCTCAAAAATTCATGTCCACATCAGTAGGAGACAGGGTCAGCGTCACCTGCAAGGCCAGTCAGAATGTGG
GTACTAATGTAGCCTGGTATCAACAGAAACCAGGGCAATCTCCTAAAGCACTGATTTACTCGGCATCCTACCGGTACAGTGGAGTCCCTGATC
GCTTCACAGGCAGTGGATCTGGGACAGATTTCACTCTCACCATCAGCAATGTGCAGTCTGAAGACTTGGCAGAGTATTTCTGTCAGCAATAT
AACAGCTATCCTTACACGTTCGGAGGGGGGACCAAGCTGGAAATAAAACGGGCTGATGCTGCACCAACT

E. NRC-2914 (HBHA)

>NRC-2914- IgG2a, GenBank accession number# MW861697, length: 1-405 bps, CDS 1-405

ATGGGATGGAGCTATATCATCCTCTTCTTGGTAGCAACAGCTACAGGTGTCCACTCTCAGGTCCAACTGCAGCAGCCTGGGGCTGAGTTTGT
GAAGCCTGGGACTTCAGTGAAGGTGTCCTGTAAGACTTCTGGCTACAACTTCACCAGACACTGGATAAACTGGGTGAAGCTGAGGCCTGGA
CAAGGCCTTGAGTGGATTGGAGATATTTATCCTGGTAGTGGTGAGACTAATTACAATGGGAAGTTCGAAAACAAGGCCACACTGACTGTAGA
CATATCCTCCAGCACAGCCTACCTGCAACTCAGCAGCCTGGCATCTGAGGACTCTGCTCTCTATTACTGTGCAAGATATGATTACGACGTTGA
CTACTGGGGCCAAGGCACCGCTCTCACAGTCTCCTCA

>NRC-2914- Ig light chain, GenBank accession number# MW861705, length: 1-440 bps, CDS 38-440

ATGGGGATTGTCATTGCAGCCAGGACTCAGCATGGACATGAGGACCCCTGCTCAGTTTCTTGGAATCTTGTTGCTCTGGTTTCCAGGTATCA
AATGTGACATCAAGATGACCCAGTCTCCATCTTCCATTTATGCATCTCGAGGAGAGAGAGTCACTATCACTTGCAAGGCGAGTCAGGACATTA
ATAGCTATTTAAGCTGGATCCAGCAGAACCCAGGGAAATCTCCTAAGACCCTGATCTATCGTGCAAGCAGATTGGTAGATGGGGTCCCATCAA
GGTTCAGTGGCAGTGGATCTGGGCAAGATTATTCTCTCACCATCAGCAGCCTGGAATATGAAGATATGGGAATTTATTATTGTCAACAGTATGA
TGAGTTTCCGCTCACGTTCGGTGGTGGGACCAAGCTGGAGCTGAAACGGGCTGATGCTGCACCAACTG

F. NRC-49679 (DnaK)

>NRC-49679- IgG1, GenBank accession number# MW861698, length: 1-604 bps, CDS 60-604

ATGGGGAAAAAAAATACGATCAGCATCCTCTCCACAGACACTGAAAACTCTGACTCACAATGGAAAGGCACTGGATCTTTCTCTTCCTGTTTT
CAGTAACTGCAGGTGTCCACTCCCAGGTCCACCTTCAGCAGTCTGGGGCTGAACTGGTAAAACCTGGGGCCTCAGTGAAGATGTCCTGCA
AGGCTTCTGGCTACACCTTTACTACCTACTGGATGCACTGGGTAAAACAGAGGCCTGGACAGGGTCTGGAATGGATTGGATACATTAATCCTA
GCACTGGTTATACTGAGTACAATCAGAAGTTCAAGGACAAGGCCACATTGACTGCAGACAAATCCTCCAGCACAGCCTACATGGAACTGAGC
AGCCTGACATCTGAGGACTCAGCAGTCTATTACTGTGCAAGAAACTCCTGGTTTGCTTACTGGGGCCAAGGGACTCTGGTCACTGTCTCTG
CAGCCAAAACGACACCCCCATCTGTCTATCCACTGGCCCCTGGATCTGCTGCCCAAACTAACTCCATGGTGACCCTGGGATGCCTGGTCAA
GGGCTATTTCCCTGAGCCAGTGACAGTGACCTGGAACTCTGGATCCCTGTCCAG

>NRC-49679- Igκ, GenBank accession number# MW861706, length: 1-452 bps, CDS 37-452

ATGGGGACTTTTGACTCACCATATCAAGTTCGCAGAATGAGGTTCTCTGCTCAGCTTCTGGGGCTGCTTGTGCTCTGGATCCCTGGATCCAC
TGCAGATATTGTGATGACGCAGGCTGCATTCTCCAATCCAGTCACTCTTGGAACATCAGCTTCCATCTCCTGCAGGTCTAGTAAGAGTCTCCT
ACATAGTAATGGCATCACTTATTTGTATTGGTATCTGCAGAAGCCAGGCCAGTCTCCTCAGCTCCTGATTTATCAGATGTCCAACCTTGCCTCA
GGAGTCCCAGACAGGTTCAGTTGCAGTGGGTCAGGAACTGATTTCACACTGAGAATCAGCAGAGTGGAGGCTGAGAATGTGGGTGTTTATT
ACTGTGCTCAAAATCTAGAACTTCCGTGGACGTTCGGTGGAGGCACCAAGCTGGAAATCAAACGGGCTGATGCTGCACCAAC

G. NRC-50100 (DnaK)

>NRC-50100- IgG1, GenBank accession number# MW861699, length: 1-604 bps, CDS 60-604

ATGGGGAAAAAAAATACGATCAGCATCCTCTCCACAGACACTGAAAACTCTGACTCACAATGGAAAGGCACTGGATCTTTCTCTTCCTGTTTT
CAGTAACTGCAGGTGTCCACTCCCAGGTCCACCTTCAGCAGTCTGGGGCTGAACTGGTAAAACCTGGGGCCTCAGTGAAGATGTCCTGCA
AGGCTTCTGGCTACACCTTTACTACCTACTGGATGCACTGGGTAAAACAGAGGCCTGGACAGGGTCTGGAATGGATTGGATACATTAATCCTA
GCACTGGTTATACTGAGTACAATCAGAAGTTCAAGGACAAGGCCACATTGACTGCAGACAAATCCTCCAGCACAGCCTACATGGAACTGAGC
AGCCTGACATCTGAGGACTCAGCAGTCTATTACTGTGCAAGAAACTCCTGGTTTGCTTACTGGGGCCAAGGGACTCTGGTCACTGTCTCTG
CAGCCAAAACGACACCCCCATCTGTCTATCCACTGGCCCCTGGATCTGCTGCCCAAACTAACTCCATGGTGACCCTGGGATGCCTGGTCAA
GGGCTATTTCCCTGAGCCAGTGACAGTGACCTGGAACTCTGGATCCCTGTCCAG

>NRC-50100- Igκ, GenBank accession number# MW861707, length: 1-452 bps, CDS 37-452

ATGGGGACTTTTGACTCACCATATCAAGTTCGCAGAATGAGGTTCTCTGCTCAGCTTCTGGGGCTGCTTGTGCTCTGGATCCCTGGATCCAC
TGCAGATATTGTGATGACGCAGGCTGCATTCTCCAATCCAGTCACTCTTGGAACATCAGCTTCCATCTCCTGCAGGTCTAGTAAGAGTCTCCT
ACATAGTAATGGCATCACTTATTTGTATTGGTATCTGCAGAAGCCAGGCCAGTCTCCTCAGCTCCTGATTTATCAGATGTCCAACCTTGCCTCA
GGAGTCCCAGACAGGTTCAGTTGCAGTGGGTCAGGAACTGATTTCACACTGAGAATCAGCAGAGTGGAGGCTGAGAATGTGGGTGTTTATT
ACTGTGCTCAAAATCTAGAACTTCCGTGGACGTTCGGTGGAGGCACCAAGCTGGAAATCAAACGGGCTGATGCTGCACCAAC

H. NRC-50101 (KatG)

>NRC-50101- IgM, GenBank accession number# MW861700, length: 1-550 bps, CDS 35-550

ATGGGGATCTCCTCACTAGAGCCCCCATCAGAGCATGGCTGTCCTGGTGCTGTTCCTCTGCCTGGTTGCATTTCCAAGCTGTGTCCTGTCC
CAGGTGCAGCTGAAGGAGTCAGGACCTGGCCTGGTGGCGCCCTCACAGAGCCTGTCCATCACTTGCACTGTCTCTGGGTTTTCATTAACC
AGCTATGGTGTACACTGGGTTCGCCAGCCTCCAGGAAAGGGTCTGGAGTGGCTGGGAGTAATATGGGCTGGTGGAAGCACAAATTATAATT
CGGCTCTCATGTCCAGACTGAGCATCAGCAAAGACAACTCCAAGAGCCAAGTTTTCTTAAAAATGAACAGTCTGCAAACTGATGACACAGCC
ATGTACTACTGTGCCAGAGATGGGTTTGCTTACTGGGGCCAAGGGACTCTGGTCACTGTCTCTGCAGAGAGTCAGTCCTTCCCAAATGTCTT
CCCCCTCGTCTCCTGCGAGAGCCCCCTGTCTGATAAGAATCTGGTGGCCATGGGCTGCCTGGCCCGGGACTTCCTGCCCAGCACCATTTC
CTTC

>NRC-50101- Igκ, GenBank accession number# MW861708, length: 1-464 bps, CDS 52-464

ATGGGGACTGATCAGTCTCCTCAGGCTGTCTCCTCAGGTTGCCTCCTCAAAATGAAGTTGCCTGTTAGGCTGTTGGTGCTGATGTTCTGGAT
TCCTGCTTCCAGCAGTGATGTTGTGATGACCCAAACTCCACTCTCCCTGCCTGTCAGTCTTGGAGATCAAGCCTCCATCTCTTGCAGATCTA
GTCAGAGCCTTGTACACAGTAATGGAAACACCTATTTACATTGGTACCTGCAGAAGCCAGGCCAGTCTCCAAAGCTCCTGATCTACAAAGTTT
CCAACCGATTTTCTGGGGTCCCAGACAGGTTCAGTGGCAGTGGATCAGGGACAGATTTCACACTCAAGATCAGCAGAGTGGAGGCTGAGG
ATCTGGGAGTTTATTTCTGCTCTCAAAGTACACATGTTCCTCCGACGTTCGGTGGAGGCACCAAGCTGGAAATCAAACGGGCTGATGCTGCA
CCAAC

I. NRC-50703 (Mpt64)

>NRC-50703- IgG1, GenBank accession number# MW861701, length: 1-443 bps, CDS 1-443

GACGTGATGCTCGAGGAGTCTGGGGGAGGCTTAGTGAAGCTTGGAGGGTCCCTGAAACTCTCCTGTGCAGCCTCTGGATTCAGTTTCAGT
AGCCATTACATGTCTTGGGTTCGCCAGACTCCGGAGAAGAGGCTGGAGTGGGTCGCAACCATTAGTAATGATGGTGGCAGCACCTACTATC
CAGACAGTGTGAAGGACCGATTCACCATCTCCAGAGACAATGCCAAGAACACCCTGTACCTGCAAATGAGTAGTCTGAATTCTGAGGACAC
AGCCGTGTATTATTGTGCAAGGTATAGGTACTTTGATTTTTGGGGCCAAGGCACCACTCTCACAGTCTCCTCAGCCAAAACGACACCCCCAT
CTGTCTATCCACTGGCCCCTGGATCTGCTGCCCAAACTAACTCCATGGTGACCCTGGGATGCCTGGTCAAGGGCTATTT

>NRC-50703- Igκ, GenBank accession number# MW861709, length: 1-588 bps, CDS 178-588

ATGGGGTGGTTTGCCTTAGTTAACAGACCATGTGTTTATAAGACAAGAACTGCGTGTCCTATATCTTGATATTTGCATACTTCATCTTCAGTAGT
CACAAATATCTCACAGCTGTTTTAAAGCGATGTACTTAGGAGAAGAGCAGTAATTAGCCAGGGAACAAAATTCAAATACACAATGGATTTTCAG
GTGCAGATTTTCAGCTTCCTGCTAATCAGTATCTCAGTTGTAATGTCCAGAGGAGAAAATGTGCTCACCCAGTCTCCAGCAATCATGTCTGCA
TCTCTAGGGGAGAAGGTCACCATGAGTTGCAGGGCCAGTTCAAGTGCAAATTACATGTACTGGTACCAGCAGAAGTCAGATGCCTCCCCCA
CACTATTGATTTATTACACATCCAACCTGGCTCCTGGAGTCCCAAGTCGCTTCAGTGGCAGTGGGTCTGGGAACTCTTATTCTCTCACAATCA
GCAGCATGGAGGGTGAAGATGTTGCCACTTATTACTGCCAGCAGTTTACTACTTCCCCATCCATGTACACGTTCGGAGGGGGGACCAAGCT
GGAAATAAAACGGGCTGATGCTGCACCAACT

J. NRC-2410 (PhoS1/PstS1)

> NRC-2410- IgG1, GenBank accession number# MW812375, length: 1-623 bps, CDS 60-623

ATGGGGAAACAGCATATGATCAGTGTCCTCTCCAAAGTCCTTGAACATAAACTCTAACCATGGAATGGACCTGGGTCTTTCTCTTCCTCCTGT
CAGTGACTGCAGGTGTCCACTCCCAGGTTCAGCTGCAGCAGTCTGGAGCTGAACTGATGAAGCCTGGGGCCTCAGTGAAGATATCCTGCA
AGGCAACTGGCTACACATTCAGTGGTTACTGGGTAGAGTGGGTAAAGCAGAGGCCTGGACATGGCCTTGAGTGGATTGGAGAGATTTTACC
TGGAAGAGTTAGCACTAATTACAATGAGAAGTTCAAGGCCAAGGCCACATTCACTGCAGATACATCCTCCAACACAGCCTACATGCAACTCA
GCAGCCTGACATCTGAGGACTCTGCCGTCTATTACTGTGCAAGATTCAAGAATTACTACGGTAGTAGTTACAACTACTTTGACTACTGGGGCC
AAGGCACCACTCTCACAGTTTCCTCAGCCAAAACGACACCCCCATCTGTCTATCCACTGGCCCCTGGATCTGCTGCCCAAACTAACTCCATG
GTGACCCTGGGATGCCTGGTCAAGGGCTATTTCCCTGAGCCAGTGACAGTGACCTGGAACTCTGGATCCCTG

> NRC-2410- Igκ, GenBank accession number# MW812376, length: 1-454 bps, CDS 37-454

ATGGGGACTCAAGACTTTTTGTATCAAGTTCTCAGAATGAGGTGCCTAGCTGAGTTCCTGGGGCTGCTTGTGCTCTGGATCCCTGGAGCCAT
TGGGGATATTGTGCTGACTCAGGCTGCACCCTCTGTGCCTGTCACTCCTGGAGAGTCACTTTCCATCTCCTGCAGGTCTAGTAAGAGTCTC
CTGCATAGTAATGGCAACACTTACTTGTATTGGTTCCTACAGAGGCCAGGCCAGTCTCCTCAACTCCTGATATATCGGATGTCCAACCTTGCC
TCAGGAGTCCCAGACAGGTTCAGTGGCAGTGGGTCAGGAACTGCTTTCACACTGAGAATCAGTAGAGTGGAGGCTGAGGATGTGGGTGTT
TATTACTGTATGCAACATCTAGAATATCCGTACACGTTCGGAGGGGGGACCAAGCTGGAAATAAAACGGGCTGATGCTGCACCAACTG

K. NRC-13810 (SodA)

> NRC-13810- IgG1, GenBank accession number# MW812377, length: 1-643 bps, CDS 72-643

ATGGGAGACGCACAACCCTGGACTCACAAGTCTTTCTCTTCAGTGACAAACACAGAAATAGAACATTCACCATGTACTTGGGACTGAACTGT
GTATTCATAGTTTTTCTCTTAAAAGGTGTCCAGAGTGAAGTGAGGCTTGAGGAGTCTGGAGGAGGCTTGGTGCTACCTGGAGGATCCATGAA
ACTCTCCTGTGTTGCCTCTGGATTCACTTTCAATAACTACTGGATGAACTGGGTCCGCCAGTCTCCAGAGAAGGGGCTTGAGTGGGTTGCT
GAAATTAGATTGAAATCTAATAATTATGCAACACATTATGCGGAGTCTGTGAAAGGGAGGTTCACCATCTCAAGAGATGATTCCAAAGGTGGTG
TCTACCTGCAAATGAACAACTTAAGAGCTGAAGACACTGGCATTTATTACTGTACCAGGGAGGCCAACAGGGGGTTTGCTTACTGGGGCCAA
GGGACTCTGGTCACTGTCTCTGCAGCCAAAACGACACCCCCATCTGTCTATCCACTGGCCCCTGGATCTGCTGCCCAAACTAACTCCATGG
TGACCCTGGGATGCCTGGTCAAGGGCTATTTCCCTGAGCCAGTGACAGTGACCTGGAACTCTGGATCCCTGTCCAGCGGAAGGGCGAATT
C

> NRC-13810- Igκ–1, GenBank accession number# MW812378, length: 1-499 bps, CDS 86-499

ATGAAATAATTTTATAACAGCCCAGGCTTCTTTAAGGGCAGCTGCCAGGATCCTAAGAAAGCATCCTCTCTTCCAGCTCTCAGAGATGGAGAC
AGAGACACTCCTGCTATGGGTGCTACTGCTCTGGGTTCCAGGTTCCACAGGTAAAATTGTGCTGACCCAATCTCCAGCTTCTTTGGCTGTGT
CTCTAAGGCAGAGGGCCACCATATCCTGCAGAGCCAGTGAAAGTGTTGATAGTTATGGCAAAAGTTTTATGCACTGGTACCAGCAGAAATCA
GGACAGCCACCCAAACTCCTCATCTATCGTGCATCCAACCTAGAATCTGGGGTCCCTGCCAGGTTCAGTGGCAGTGGGTCTAGGACAGACT
TCACCCTCACCATTGATCCTGTGGAGGCTGATGATGCTGCAACCTATTACTGTCAGCAAAATTATGAGGCTCCTCGGACGTTCGGTGGAGGC
ACCAAGCTGGAAATCAAACGGGCTGATGCTGCACCAACT

> NRC-13810- Igκ–2, GenBank accession number# MW812379, length: 1-435 bps, CDS 22-435

CCTCTCTTCCAGCTCTCAGAGATGGAGACAGACACACTCCTGTTATGGGTACTGCTGCTCTGGGTTCCAGGTTCCACTGGTGACATTGTGC
TGACACAGTCTCCTGCTTCTTTGGCTGTGTCTCTAAGGCAGAGGGCCACCATATCCTGCAGAGCCAGTGAAAGTGTTGATAGTTATGGCAAA
AGTTTTATGCACTGGTACCAGCAGAAATCAGGACAGCCACCCAAACTCCTCATCTATCGTGCATCCAACCTAGAATCTGGGGTCCCTGCCAG
GTTCAGTGGCAGTGGGTCTAGGACAGACTTCACCCTCACCATTGATCCTGTGGAGGCTGATGATGCTGCAACCTATTACTGTCAGCAAAATT
ATGAGGCTCCTCGGACGTTCGGTGGAGGCACCAAGCTGGAAATCAAACGGGCTGATGCTGCACCAACT

L. NRC-49680 (KatG)

> NRC-49680 IgM, GenBank accession number# MW812380, length: 1-556 bps, CDS 38-556

ATGGGGCATCCTCTTCTCATAGAGCCTCCATCAGAGCATGGCTGTCCTGGGGCTGCTTCTCTGCCTGGTGACGTTCCCAAGCTGTGTCCTG
TCCCAGGTGCAGCTGAAGGAGTCAGGACCTGGCCTGGTGGCGCCCTCACAGAGCCTGTCCATCACATGCACTGTCTCAGGGTTCTCATTA
ACCGACTATGGTGTAAGCTGGATTCGCCAGCCTCCAGGAAAGGGTCTGGAGTGGCTGGGAGTAATATGGGGTGGTGGAAGCACATACTATA
ATTCAGCTCTCAAATCCAGACTGAGCATCAGCAAGGACAACTCCAAGAGCCAAGTTTTCTTAAAAATGAACAGTCTGCAAACTGATGACACA
GCCATGTACTACTGTGCCAAACATGGTAACTTTGCTTACTGGGGCCAAGGGACTCTGGTCACTGTCTCTGCAGAGAGTCAGTCCTTCCCAAA
TGTCTTCCCCCTCGTCTCCTGCGAGAGCCCCCTGTCTGATAAGAATCTGGTGGCCATGGGCTGCCTGGCCCGGGACTTCCTGCCCAGCAC
CATTTCCTTC

> NRC-49680- Igκ, GenBank accession number# MW812381, length: 1-465 bps, CDS 56-465

ATGGGGACTTATGATAATAGCAGTAATTAGCTAGGGACCAAAATTCAAAGGAAAAATGGATTTTCAGGTGCAGATTTTCAGCTTCCTGCTAATC
AGTGCCTCAGTCATAATGTCCAGAGGACAAATTGTTCTCACCCAGTCTCCAGCAATCATGTCTGCATCTCTAGGGGAACGGGTCACCATGAC
CTGCACTGCCAGCTCAAGTGTAAGTTCCAGTTACTTGCACTGGTACCAGCAGAAGCCAGGATCCTCCCCCAAACTCTGGATTTATAGCACAT
CCAACCTGGCTTCTGGAGTCCCAGCTCGCTTCAGTGGCAGTGGGTCTGGGACCTCTTACTCTCTCACAATCAGCAGCATGGAGGCTGAAG
ATGCTGCCACTTATTACTGCCACCAGTATCATCGTTCCCCGTGGACGTTCGGTGGAGGCACCAAGCTGGAAATCAAACGGGCTGATGCTGC
ACCAAC

M. NRC-2894 (GroES)

> NRC-2894- IgG2a, GenBank accession number# MW812373, length: 1-678 bps, CDS 70-678

ATGGGGAGGCAGAGAACTTTAGCCCTGTCTTCTTTTTTAGTGTTCAGCACTGACAATATGACATTGAACATGCTGTTGGGGCTGAAGTGGGT
TTTCTTTGTTGTTTTTTATCAAGGTGTGCATTGTGAGGTGCAGCTTGTTGAGTCTGGTGGAGGATTGGTGCAGCCTAAAGGGTCATTGAAACT
CTCATGTGCAGCCTCTGGATTCACCTTCAAAACCTACGCCATGAACTGGGTCCGCCACACTCCAGGAAAGGGTTTGGAATGGGTTGCTCGC
ATAAGAAGTAAAAGTAATAATTTTGCAACATATTATGCCGATTCAGTGAAAGACCGGTTCACCATCTCCAGAGATGATTCACAAAGCATGCTCTA
TCTGCAAATGAACAACTTGAAAACTGAGGACACAGCCATGTATTACTGTGTGAAACTAACTAACGGCTACTTTGACTCCTGGGGCCAAGGCA
CCACTCTCACAGTCTCCTCAGCCAAAACAACAGCCCCATCGGTCTATCCACTGGCCCCTGTGTGTGGAGATACAACTGGCTCCTCGGTGAC
TCTAGGATGCCTGGTCAAGGGTTATTTCCCTGAGCCAGTGACCTTGACCTGGAACTCTGGATCCCTGTCCAGTGGTGTGCACACCTTCCCA
GCTGTCCTGCAGTCTGACCTCTACACCCTCAGC

> NRC-2894- Igκ, GenBank accession number# MW812374, length: 1-450 bps, CDS 51-450

ATGGGGATACACACTCCAACAGTCATTCTTGGTCAGGAGACGTTGTAGAAATGAGACCGTCTATTCAGTTCCTGGGGCTCTTGTTGTTCTGG
CTTCATGGTGTTCAGTGTGACATCCAGATGACACAGTCTCCATCCTCACTGTCTGCATCTCTGGGAGGCAAAGTCACCATCACTTGCAAGGC
AAGCCAAGACATTAACAATTATATAGCTTGGTACCAACACAAGCCTGGAAAAGGTCCTAGGCTGCTCATACATGACACATCAACATTACAGCC
AGGCATCCCATCAAGGTTCAGTGGAAGTGGGTCTGGGAGAGATTATTCCTTCAGCATCAGCAACCTGGAGCCTGAAGATATTGCAACTTATT
ATTGTCTACAGTATGATAATCTTCGGACGTTCGGTGGAGGCACCAAGGTGGAAATCAAACGGGCTGATGCTGCACCAACTG
